# Assessment of nanopore RNA modification calling in human cell lines and synthetic systems

**DOI:** 10.1186/s13059-026-04096-w

**Published:** 2026-05-07

**Authors:** Neda Ghohabi Esfahani, Andrew J. Stein, Stuart Akeson, Talia Tzadikario, Connor Powell, Pooria Daneshvar Kakhaki, Miten Jain

**Affiliations:** 1 Department of Bioengineering, Northeastern University, Boston, MA USA; 2 Department of Electrical & Computer Engineering, Northeastern University, Boston, MA USA; 3 Department of Physics, Northeastern University, Boston, MA USA; 4 Khoury College of Computer Sciences, Northeastern University, Boston, MA USA

**Keywords:** Direct RNA sequencing, RNA modification, Nanopore sequencing, Modification calling performance

## Abstract

**Background:**

Nanopore technology enables the direct sequencing of intact RNA molecules allowing for the detection of native chemical modifications. In 2024, Oxford Nanopore Technologies updated direct RNA sequencing from RNA002 to RNA004 platform as well as releasing an improved basecaller (Dorado) capable of de novo detection of eight RNA modifications. We compare RNA002 and RNA004 platforms for poly(A) RNA from GM12878 and HEK293 cell lines and evaluate Dorado-based RNA modification calling.

**Results:**

We compute U-to-C mismatches, previously used to identify putative pseudouridine sites, and run m6anet for identifying putative N6-methyladenosine sites. We find that Dorado identifies global and site-specific differences when compared to RNA002 methods. We examine eight RNA modifications detected by Dorado for Nanopore direct RNA sequencing data and propose an analysis strategy for curating RNA modification predictions, including thresholds for read coverage and modification occupancy, canonical RNA-based false positive correction, and comparison with orthogonal information. When comparing modification sites called by Dorado versus those documented by orthogonal datasets, we note significant discordance and we document disagreements between our results and orthogonal datasets.

**Conclusions:**

The transition from RNA002 to RNA004 substantially improves sequencing accuracy and modification calling. However, Nanopore direct RNA sequencing-based RNA modification detection requires careful validation. We recommend combining Nanopore direct RNA sequencing with orthogonal methods and appropriate filtering strategies for increased confidence in modification calls.

**Graphical abstract:**

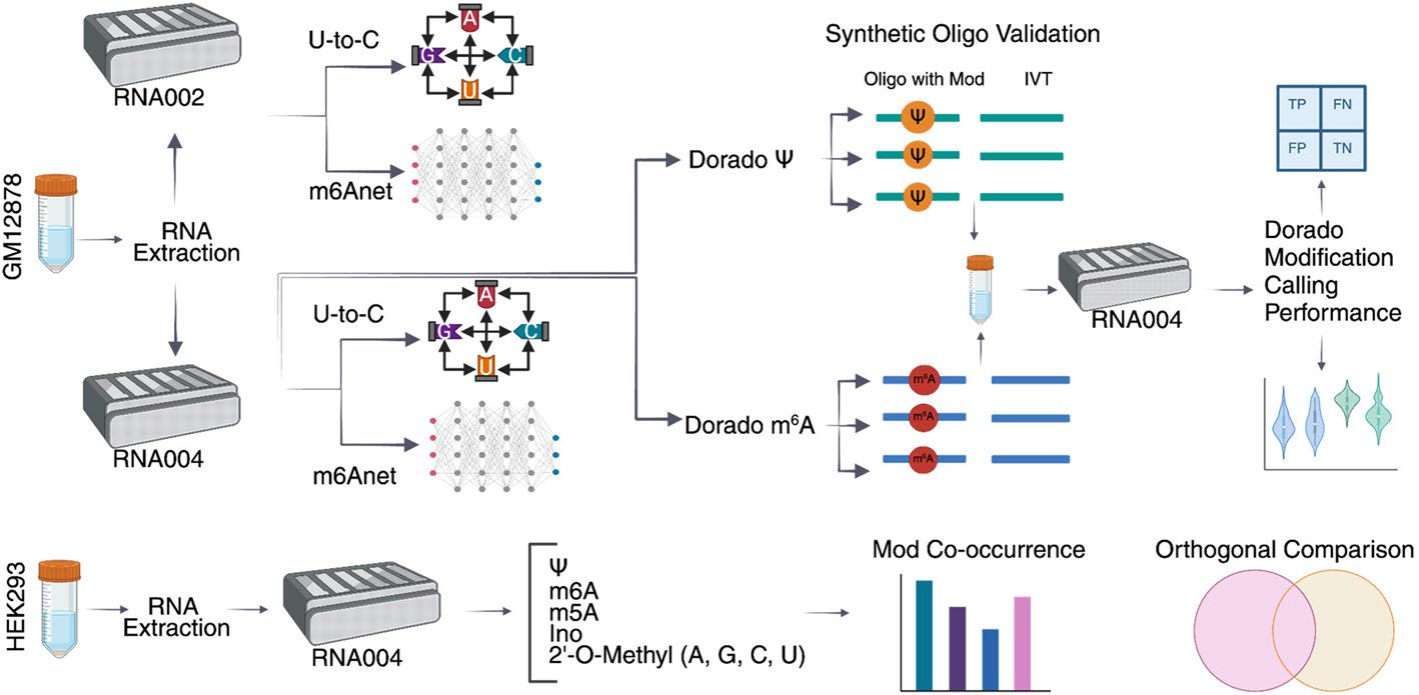

**Supplementary Information:**

The online version contains supplementary material available at 10.1186/s13059-026-04096-w.

## Background

Nanopore direct RNA sequencing (DRS) is the only technology that allows for long readouts of native RNA molecules [1, 2]. Briefly, DRS involves the enzymatic regulation of an RNA strand translocating through a biological nanopore under applied voltage. Ionic current changes corresponding to the nucleotide composition in the nanopore sensor are recorded and interpreted (basecalled) into RNA sequences by deep learning algorithms. An advantage of DRS is that the native RNA molecules are read, rather than a synthetic DNA copy [2]. Another key feature of DRS is the direct, simultaneous detection of RNA modifications at a single-molecule level [1, 3–7]. RNA modifications are chemical alterations to ribonucleotides, with over 170 unique types identified to date [8]. These modifications are documented across diverse classes of RNA [9], impacting translation [10], stability [11], molecular structure [12], and cancer [13].

As with all machine learning models, the basecaller can make errors. Nucleotide modifications have been identified as one potential source of these errors. For example, uridine modifications were semi-systematically miscalled as a cytosine in data generated using RNA002 chemistry and basecalled using Guppy [5, 6]. Alternatively, ionic current models were developed to interpret specific modifications. For example, m6anet [14] differentiated N6-methyladenosine (m^6^A) from adenosine using ionic current and basecalled sequence data. Both the miscall error techniques and ionic current models for modification calling required continued development as DRS technology evolved.

The majority of DRS studies used the RNA002 sequencing platform, which was available from 2019 to 2024. In 2024, ONT updated its DRS sequencing platform to RNA004 [15]. This update included a new RNA-specific Nanopore, updated biochemistry, and new software. This meant that the traditional RNA modification identification techniques from RNA002 were no longer directly applicable, and the strategies required validation before being applied to RNA004 data. With RNA004 platform, ONT released an updated basecaller (Dorado [16]), that can also call eight RNA modifications [17] (pseudouridine (Ψ), m^6^A, 5-methylcytosine (m^5^C), inosine (I), and four 2'-O-methylation (2'-O-Methyl) modifications [16]). None of these modification callers were supported with independent verification by the research community at the time of their release. Orthogonal comparison techniques are crucial as modification-calling strategies advance. These validation strategies include true negative in vitro transcription (IVT) derived RNA [4, 18], enzymatic knockdowns and knockouts [19, 20], liquid chromatography with tandem mass spectrometry (LC–MS/MS) [19, 21], and sequencing of synthetic oligonucleotides with and without modifications [22].

In this study, we compared DRS RNA002 platform with DRS RNA004 platform chemistry. We also RNA004 platform-based DRS modification calls with orthogonal methods. We used RNA extracted from the GM12878 cell line and sequenced it using both chemistries. Our analysis documented improvements in alignment identity, insertion and deletion rates, and base substitution rates between the two chemistries. We also compared RNA004 modification calling methods (Dorado-based) with RNA002 modification calling methods (U-to-C mismatches, m6anet). To minimize false positives, we first applied a read coverage cutoff, followed by filtering based on modification occupancy (defined as the proportion of reads called as modified at a given position). We also used an IVT-controlled modification occupancy rate for each position to subtract from the RNA004 Dorado calls, producing a subset of higher confidence putative modification sites [23]. For a small subset of sequence contexts, we analyzed synthetic oligonucleotides both with and without a modification in the same sequence context as putative modifications in our data for Ψ and m^6^A. These synthetic controls provided validation for a set of modification calls by the novel RNA004-based modification callers. Further, we sequenced three technical replicates of RNA extracted from HEK293, and investigated the occurrence of the four modifications Ψ, m^6^A, m^5^C and inosine as called by Dorado. We compared DRS results for each respective modification with existing orthogonal datasets for HEK293 cells.

## Results

### RNA004 chemistry has improved sequencing performance compared with RNA002 chemistry

The GM12878 cell line derived RNA002 data from Workman et al. were generated using 30 MinION flow cells and had 9,729,412 pass reads (out of 13.0 M reads total) with a read N50 of 1.36 kb. From these data, 9.4 M reads aligned (96.8% of total reads) aligned to the GRCh38 (hg38) reference genome [24], spanning 12,212 Gencode annotated genes with > 10 aligned reads. By comparison, The GM12878 cell derived RNA004 data that we generated using one PromethION flow cell had 26,820,281 reads with a read N50 of 1.88 kb. From these data, 16.6 M reads (61.94% of total reads) aligned to the reference genome and spanned 13,773 Gencode annotated genes with the same filtering criteria (see “Methods"). Despite differences in total read depth and flow cell configuration, median per-gene coverage was comparable (124 and 121 reads per gene for RNA002 and RNA004, respectively), and per-gene expression levels were highly correlated (Pearson r = 0.80, *n* = 11,562 shared genes; Additional file 1: Fig. S1.C and S1.D).

**Fig. 1:**
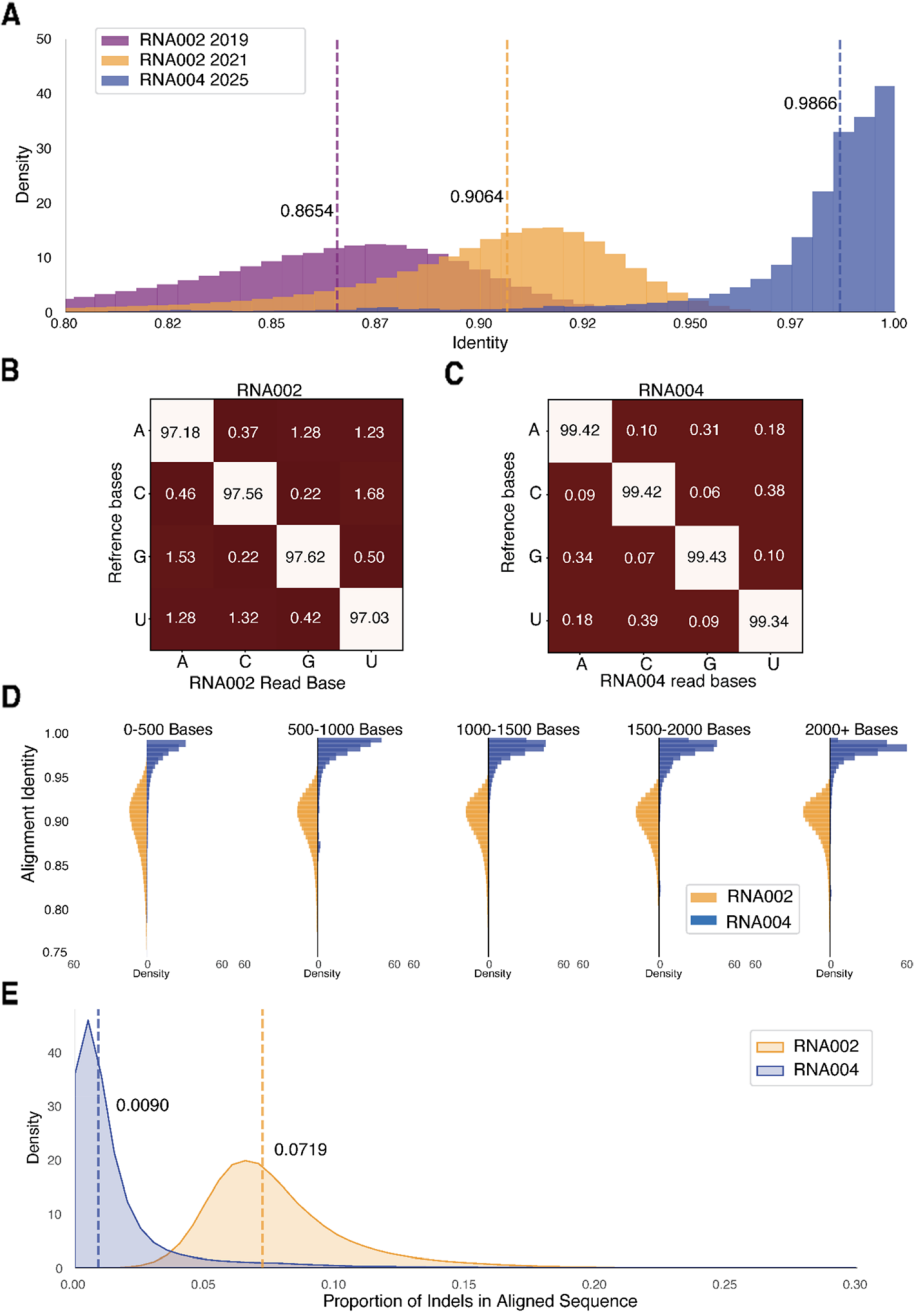
Comparison of RNA002 and RNA004 direct RNA sequencing performance. **A** Aligned read identity was calculated as a percentage of total matches over the sum of total matches, mismatches, insertions, and deletions in the aligned region. This calculation was repeated for multiple sequencing chemistries across multiple versions of basecallers all for the same extraction of GM12878 RNA. The median alignment identity for each is shown with a dashed line. **B** RNA002 base-specific substitution matrix calculating the rate at which, given a reference base, each possible canonical nucleotide was observed in aligned regions of reads. **C** RNA004 base-specific substitution matrix using the same method as the RNA002 base-substitution matrix. **D** Read identity histograms for RNA002 and RNA004 binned by aligned read length in 500 aligned nucleotide buckets. Aligned read length was calculated as the genomic distance between the first aligned base of the read and the last aligned base of the read. **E** Insertion and deletion proportion for RNA002 and RNA004 calculated as the number of insertions over total aligned bases and deletions over total aligned bases, respectively, median shown with dashed lines

We compared the alignment identity to the hg38 reference genome between RNA002 and RNA004. RNA002 data, basecalled with Guppy v6.38, achieved a median identity of 90.65% for reads with at least 200 aligned bases, while RNA004 data basecalled with Dorado v1.0 achieved a median alignment identity of 98.67% for the same filtering criteria. When compared to older generations of basecalling software, we documented a continued improvement in alignment identity over the last 5 years (Fig. 1A). When we computed the per-base substitution matrix, we noted that a reference base of C miscalled as U was the most prevalent miscall in RNA002, while a reference base of U miscalled as C was the most prevalent in RNA004. C-to-G and G-to-C were the least prevalent (Fig. 1B, C). The rate of these global miscalls decreased by a factor of approximately 3 when transitioning from RNA002 to RNA004. Our data suggested that the highest per-base-substitution rate of 1.68% for C-to-U in RNA002 had decreased to 0.38% in RNA004. To assess the impact of read length on alignment identity, we binned reads into 500-nucleotide buckets (Fig. 1D). We documented a higher variance in RNA002 alignment identity compared to RNA004, with a relatively steady median in each bucket, suggesting that length had no discernible impact on alignment identity. RNA004 showed a smaller variance, with a shift towards higher alignment identity for every bucket (Additional file 2: Table S1), but did observe a slight decrease in median alignment identity as lengths increased (Additional file 1: Fig. S1). To better understand the overall increase in accuracy for RNA004, we computed the proportion of insertions and deletions (INDELS) in the aligned sequences. RNA002 had a median of 7.19% INDELS per base, while RNA004 had a median of 0.9% INDELS per base (Fig. 1E).

Additionally, we investigated RNA poly(A) tail length estimates using nanopolish (polya) for RNA002 data and Dorado for RNA004 data (Additional file 1: Fig. S2.A). We grouped aligned reads into mitochondrial-sourced transcripts and genomic transcripts, with nuclear-sourced transcripts having an estimated poly(A) tail length median of 92.05 and 80 for RNA002 and RNA004, respectively. Mitochondrial-sourced transcript poly(A) length estimates had a median of 54.4 and 47 for RNA002 and RNA004, respectively. The preservation of the shape of the distributions between sequencing chemistries for two separate transcript types suggests that differential poly(A) tail analysis techniques employed in RNA002 experiments will yield similar results using the RNA004 sequencing chemistry. We verified that Dorado poly(A) estimates were concordant with the isoform-specific poly(A) tail length differences that we and others had previously shown [1, 2]. To that end, we computed isoform-specific polyA tail estimations for the *MZB1* gene transcripts. For the RNA004 *MZB1* transcripts, we were able to identify a significant difference in the poly(A) tail length distributions for the isoforms *MZB1-203* and *MZB1-201*. We were able to duplicate this finding using RNA002, indicating that poly(A) tail length estimation remained informative across chemistry updates (Additional file 1: Fig. S2.B). To further validate poly(A) estimation accuracy, we examined genes with previously characterized tail length distributions from Workman et al. [1, 2]. *DDX17*, identified as having among the longest poly(A) tails, showed median estimates of 238 nt (RNA002) and 161 nt (RNA004), while the ribosomal protein gene *RPS24*, known to have short poly(A) tails, showed median estimates of 65 nt (RNA002) and 55 nt (RNA004) (Additional file 1: Fig. S2.C).

Dorado additionally introduced a set of 8 RNA modification calling models. We first examined two modifications (Ψ and m^6^A) with their respective RNA002 modification calling strategies.

### Number of predicted Ψ sites vary between RNA002 and RNA004 DRS data

**Fig. 2:**
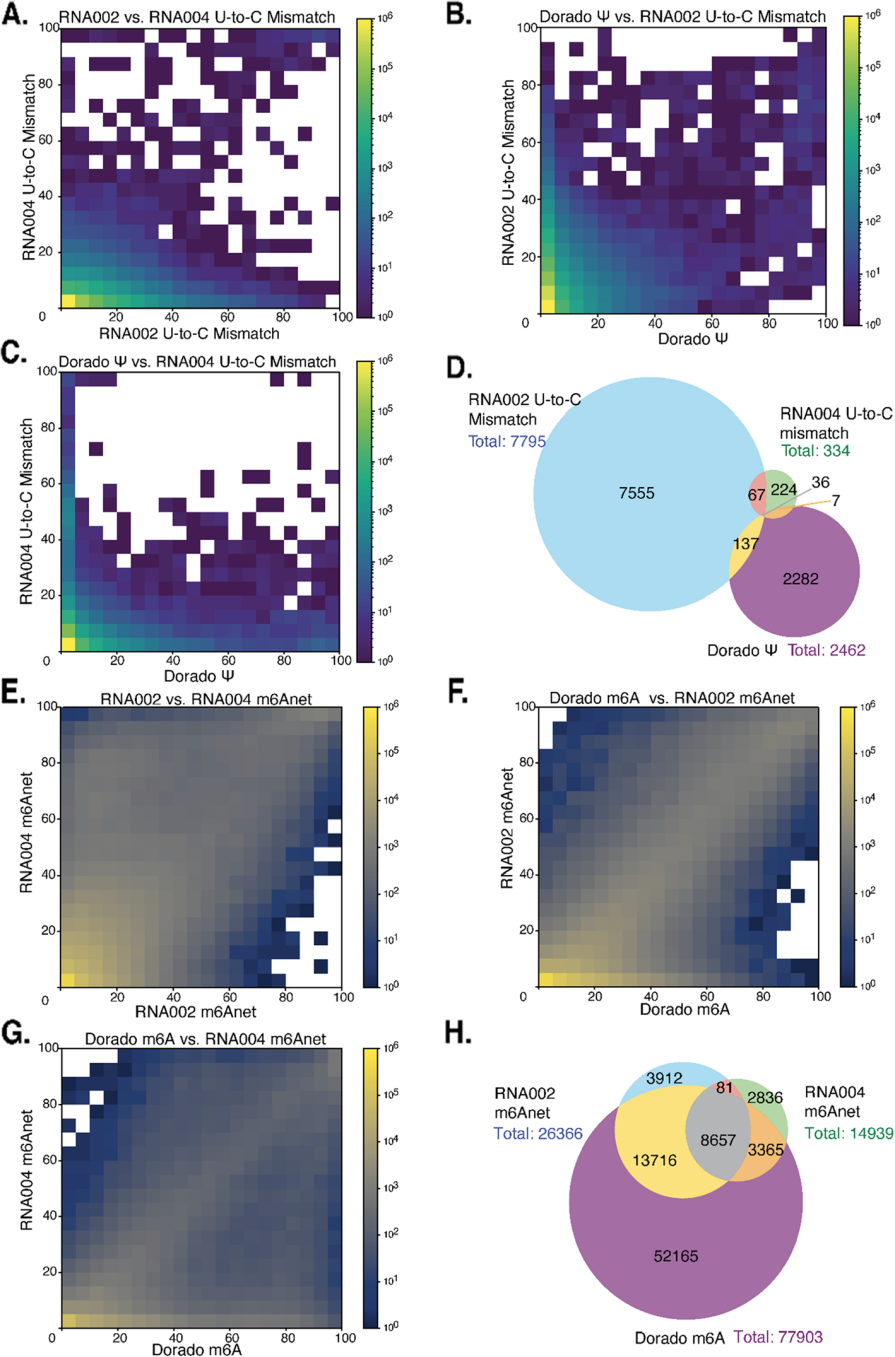
Comparison of Dorado Ψ and m^6^A models with the RNA002 modification calling strategies. **A** Comparison of U-to-C mismatch percentage between RNA002 (x-axis) and RNA004 (y-axis) for reads with valid coverage of 20. **B** Comparison of RNA002 U-to-C mismatch percentage (y-axis) and Dorado Ψ reported occupancy (x-axis) for reads with valid coverage of 20. **C** Comparison of RNA004 U-to-C mismatch percentage (y-axis) to Dorado Ψ reported occupancy (x-axis) for reads with valid coverage of 20. **D** Venn diagram showing the shared and unique sites between RNA002 U-to-C, RNA004 U-to-C and Dorado Ψ with at least 20% reported modification occupancy (or 20% U-to-C mismatch percentage) after intersecting all sites for valid coverage of 20 reads. **E** Comparison of RNA002 and RNA004 m6anet reported occupancy. **F** Comparison of RNA002 m6anet to Dorado m^6^A. **G** Comparison of RNA004 m6anet to Dorado m^6^A. **H** Shared and unique sites between RNA002 m6anet, RNA004 m6anet, and Dorado m^6^A

As U-to-C mismatches were previously used as an indicator of uridine modifications (e.g., pseudouridine [5, 6, 25]), we compared U-to-C mismatch percentages for RNA002 and RNA004 for overlapping positions between the two datasets (Fig. 2A). This analysis considered positions with at least 20 reads in coverage and involved extracting positions where the reference base was U and the observed base was C. We computed the fraction of C reads over total reads and filtered for positions with at least one U-to-C mismatch and no genomic variants (see “Methods"). The same method was used for calculating RNA004 U-to-C mismatch percentage. For 139,750 sites, RNA002 was 5 + % higher in U-to-C mismatch. For 5,274 sites, RNA004 was 5 + % higher in U-to-C mismatch. For 1,830,672 sites, the two chemistries were within 5% of each other (Additional file 1: Fig. S3). To quantify differences, we calculated the Root Mean Square Error (RMSE), which measures the average deviation between two datasets. A lower RMSE indicates closer agreement between datasets, while a higher RMSE reflects greater differences. The RMSE between RNA004 and RNA002 U-to-C mismatch percentages was 0.0447. We also assessed correlation using Pearson R^2^ and Spearman's rank correlation coefficient. The R^2^ was 0.2308, indicating that linear correlation explains only ~ 23% of the variance between the two chemistries. However, Spearman's ρ was 0.7414 (*p* < 2.2 × 10⁻^1^⁶), suggesting a moderate-to-strong monotonic relationship despite the weak linear fit. This discrepancy reflects that while RNA002 and RNA004 U-to-C mismatch percentages generally rank similarly across sites, the magnitude of mismatch often differs, with RNA002 showing systematically higher values at many positions.

We compared the U-to-C mismatch percentages for RNA002 with the RNA004 Dorado Ψ model by considering overlapping positions between the two datasets with at least 20 reads coverage (Fig. 2B). We also compared the distribution of RNA004 U-to-C mismatch percentage to RNA004 Dorado Ψ modification predictions (Fig. 2C). In both instances, the density of sites was dispersed away from the line y = x, suggesting discordance between U-to-C mismatch and the Dorado model. The RMSE between Dorado Ψ modification and RNA002 U-to-C mismatch was 0.0462, with R^2^ = 0.1482 and Spearman's *ρ* = 0.4635 (*p* < 2.2 × 10⁻^1^⁶). The RMSE between Dorado Ψ modification and RNA004 U-to-C mismatch was 0.0389, with R^2^ = 0.0901 and Spearman's *ρ* = 0.6165 (*p* < 2.2 × 10⁻^1^⁶). The low R^2^ values (< 0.15) indicate poor linear agreement between U-to-C mismatch ratios and Dorado Ψ predictions, while the moderate Spearman correlations suggest some concordance in ranking but substantial differences in absolute values. Notably, the Dorado model showed weaker linear correlation with RNA004 U-to-C mismatch (R^2^ = 0.0901) than with RNA002 (R^2^ = 0.1482).

For the intersection of valid sites in all three datasets, we used a Venn diagram to visualize the overlap of putative modification positions between datasets: RNA002 U-to-C mismatches, RNA004 U-to-C mismatches, and Dorado Ψ modification positions. We limited the analysis to sites that had valid coverage (n > = 20) in all three datasets to control for batch variability. This decreased the number of sites considered to 1,304,734. Each dataset was then filtered to include positions with at least 20% U-to-C mismatch or Dorado Ψ modification percentage. As previously stated, the genomic variants were excluded from the pool of sites (Fig. 2D). This comparison provides insights into the disagreement and agreement between these approaches. The U-to-C mismatch sites matching our criteria decreased from 7,795 positions in RNA002 to 334 sites in RNA004. This is while 2,462 of the eligible sites were detected by Dorado with at least 20% Ψ modified. Only 36 sites were shared among the three datasets. The decrease in the number of sites between RNA002 and RNA004 U-to-C mismatch indicates a sharp decrease in the miscall signal that previous studies have relied on.

### Number of predicted m^6^A sites are concordant between RNA002 and RNA004 DRS data

We compared m^6^A sites detected using m6anet for both RNA002 and RNA004 datasets. We converted the results to genomic positions and filtered for shared sites between both datasets (Fig. 2E). We also compared Dorado m^6^A calls with m6anet calls for both RNA002 dataset (Fig. 2F) and RNA004 dataset (Fig. 2G).

We used a Venn diagram to visualize the overlap of sites between datasets: RNA002 m6anet, RNA004 m6anet, and Dorado m^6^A modification positions (Fig. 2H). All three datasets were filtered to include positions with at least 20 reads in coverage and 20% modification percentage. The sights for m6anet were further filtered for 90% probability of modification. The number of sites called by m6anet decreased from 26,266 positions in RNA002 to 14,939 positions in RNA004. In contrast, 77,903 m^6^A positions were called by Dorado. 8,657 positions were shared across the three datasets. The majority of m6anet sites were agreed with Dorado, 84.86% of m6anet sites in RNA002, and 80.47% in RNA004. For the sites that agreed between m6anet and Dorado, the RMSE between the modification percentages called by either technology were 0.1347 and 0.2148, with an R^2^ of 0.5836, and 0.2353, and Spearman's *ρ* = 0.7675 (*p* < 1.0 × 10^–10^) and *ρ* = 0.5389 (*p* < 1.0 × 10^–10^), respectively for RNA002 and RNA004.

### Adjusting false positive rates for modification calls using IVT-derived canonical RNA DRS data

**Fig. 3:**
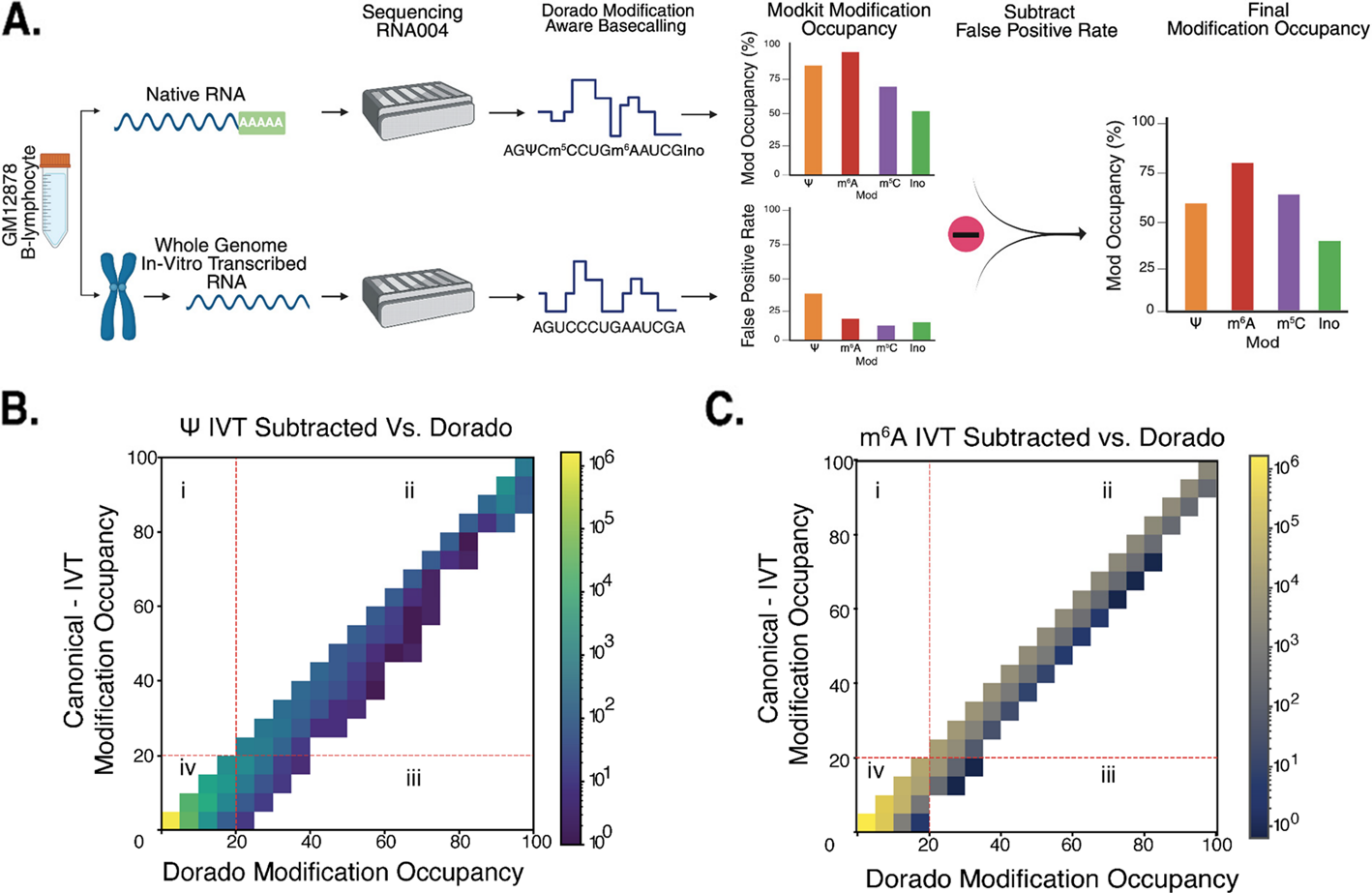
Adjusting false positive rates for modification calls using genomic DNA-based, IVT-derived canonical RNA. **A** Schematic overview of the steps involved in comparing and subtracting IVT-derived false positive rates from native RNA modification calls obtained by Dorado. We used our pre-computed 9-mer false positive rates [23] to adjust the Dorado modification calls by subtracting the 9-mer specific false positive rate from the biological and compared them against the original calls. **B** Comparison of Dorado Ψ reported modification occupancy before subtracting IVT false positive rate (x-axis) and reported modification occupancy after subtracting IVT (y-axis). The red line indicates the 20% threshold and separates the 4 regions of the 2D histogram. **C** Comparison of Dorado m^6^A reported modification occupancy before subtracting IVT false positive rate (x-axis) and reported modification occupancy after subtracting IVT (y-axis)

To improve the specificity of modification detection, we adjusted RNA004 Dorado modification calls using a whole genome IVT RNA DRS dataset [23]. Briefly, IVT RNA, composed only of canonical nucleotides, provides a biologically representative sequence context without modifications, making it an effective negative control. We used our pre-computed 9-mer false positive rates [23] to adjust the Dorado modification calls by subtracting the 9-mer specific false positive rate from the biological. We compared the false positive adjusted calls against the original calls, highlighting the area where sites fell below our 20% modification threshold (Fig. 3A). This strategy is broadly applicable for reducing the number of false positive calls. We anticipate this could also be extended to future modification calling models.

The IVT dataset [23] was generated from three PromethION DRS experiments, yielding 8,883,294 aligned RNA strand reads with a median alignment identity of 98.67%. Global false positive rates ranged between 0.0016% to 0.0086% across modification types, with pseudouridine at 0.0036% and m6A at 0.0054%. Because of sequence-specific errors, we calculated false positive rates in a 9-mer context-specific manner rather than relying solely on global rates. This approach accounts for the influence of surrounding bases on modification calling accuracy.

Each position was matched to its corresponding reference 9-mer and its IVT false-positive rate from the pre-calculated table. We subtracted IVT-reported modification occupancy from Dorado-reported occupancy to help filter sites where the model had a high false-positive rate. For biological Ψ sites, we compared the primary Dorado occupancy with the adjusted occupancy after subtracting the IVT-derived false positive rates. This approach aimed to reduce false-positive calls. Initially, 5,007 sites were detected by Dorado with at least 20 reads and a reported Ψ occupancy of 20%. After applying the IVT adjustment, this number was reduced to 3,877 sites (Fig. 3B and Table 4). In the 2D histogram, we separated the sites into four regions based on the 20% threshold used throughout our analysis. Region “i” represents the sites (*n* = 0) with < 20% original reported modification occupancy and ≥ 20% modification occupancy after subtracting IVT false positive rates, since the false-positive rate is > = 0, no sites gained estimated modification occupancy. Region “ii” contains the sites (*n* = 3,877) with ≥ 20% reported modification occupancy both before and after IVT subtraction. Region “iii” includes the sites (*n* = 1,130) with ≥ 20% original reported modification occupancy and < 20% occupancy after subtracting IVT. Region “iv” corresponds to the densest region of the histogram, containing 877,193 sites with < 20% reported modification occupancy both before and after IVT subtraction.

Similarly, for biological m6A sites, we compared the primary Dorado-reported modification occupancy with the adjusted occupancy after subtracting the IVT-derived false positive rates. The dataset originally contained 77,903 sites where Dorado had 20 reads and called m^6^A with ≥ 20% occupancy. After using the IVT correction, this number was reduced to 71,468 (Fig. 3C and Table 4). As was with Ψ, the figure is separated into four regions based on the 20% threshold used throughout our analysis. Dorado m^6^A showed a similar pattern, with region “i” (*n* = 0), region “ii” (*n* = 71,468), region “iii” (*n* = 6,435), and region “iv” (*n* = 8,333,030).

### Validation of dorado modification calls using synthetic oligonucleotides

To further evaluate the performance of Dorado modification calling for detecting Ψ and m6A at known modification sites, we designed 6 synthetic oligonucleotides with a modification at a known position and their corresponding in vitro transcribed RNA controls. Three oligos were designed for Ψ (Table 1), selected to represent extreme cases of agreement and disagreement between RNA002 U-to-C mismatch and Dorado: Ψ_1 where both methods predicted high modification (100% U-to-C, 100% Dorado), Ψ_2 where U-to-C mismatch was high but Dorado predicted low (100% U-to-C, 0% Dorado), and Ψ_3 where U-to-C mismatch was low but Dorado predicted high (0% U-to-C, 100% Dorado). Similarly, three m6A oligos were selected based on percentile ranges of occupancy difference between Dorado and m6anet (Table 2): m6A_1 (0–5th percentile), m6A_2 (95–100th percentile), and m6A_3 where both methods agreed (≥ 90% for both). For each of the synthetically modified oligonucleotides, a true negative oligonucleotide was generated using IVT (see “Methods”).

**Table 1:** Modification heuristics for RNA002 and RNA004 for selected positions with Ψ predictions

Name	U-to-C mismatch (RNA002)	Dorado occupancy (RNA004)
Ψ_1	100%	100%
Ψ_2	100%	0%
Ψ_3	0%	100%

**Table 2:** Modification heuristics for RNA002 and RNA004 for selected positions with m^6^A predictions

Name	Dorado occupancy—RNA002 m6anet occupancy	Delta percentile range
m^6^A_1	−20.91%	0-5th
m^6^A_2	23.47%	95-100th
m^6^A_3	10% (Both > = 90%)	65-70th

We performed DRS for these 12 constructs (synthetic modification-bearing oligonucleotides and canonical IVT-derived RNA) using the RNA004 platform. The raw data were basecalled using Dorado v1.0 with Ψ and m^6^A modification calling models, yielding 56,443,656 reads. Filtering for primary alignment resulted in 33,141,855 reads (57% of all aligned reads). We further filtered for full length and identification of a custom barcode sequence to ensure their identity, which resulted in 15,745,700 reads (see “Methods”). The number of full-length reads for each oligonucleotide is detailed in Table 3. ONT’s Modkit tool was run on the filtered aligned BAM file with the modification tags. We compared the modification occupancy of our known modified sites with their IVT pair. We also evaluated the performance of traditional modification detection strategies on these data.

**Table 3:** Number of full-length reads for all oligonucleotides and Dorado performance for each pair

**Oligo** **name**	**Mod reads**	**IVT reads**	**Balanced accuracy**	**Accuracy**	**Precision**	**Recall**	**F1 Score**	**AUROC**	**AUPRC**
Ψ_1	2,236,489	362,850	0.9871	0.9809	0.9995	0.9758	0.9875	0.9074	0.9850
Ψ_2	1,851,854	495,933	0.9694	0.9544	0.9997	0.9574	0.9396	0.9733	0.9934
Ψ_3	1,340,557	947,062	0.9492	0.9519	0.9930	0.9039	0.9464	0.9396	0.9615
m^6^A_1	2,071,393	159,672	0.9833	0.9794	0.9989	0.9787	0.9887	0.9924	0.9993
m^6^A_2	1,804,781	756,602	0.9545	0.9400	0.9952	0.9195	0.9559	0.9853	0.9947
m^6^A_3	1,792,189	1,080,786	0.9761	0.9725	0.9951	0.9594	0.9769	0.9847	0.9927

For the three Ψ-bearing constructs, Dorado consistently reported high occupancy at the known modified sites, ranging from 90 to 97%. In contrast, U-to-C mismatch percentages at these same sites varied substantially: 22% for Ψ_1, 4% for Ψ_2, and 2% for Ψ_3 (Additional file 1: Fig. S4A). This discrepancy highlights that while Dorado reliably detected pseudouridine across all three sequence contexts, U-to-C mismatch rates were inconsistent and substantially lower than the true modification level. In the canonical IVT pairs, both Dorado occupancy and U-to-C mismatch remained near 0%, confirming the specificity of both metrics for unmodified uridines.

We compared Dorado m^6^A calls with m6anet for these synthetic oligonucleotides (Additional file 1: Fig. S4B). We compared Dorado m^6^A calls with m6anet for these synthetic oligonucleotides. M6anet requires preprocessed data from nanopolish eventalign or an equivalent tool. To this end we used f5C eventalign, a signal realignment tool recommended by the m6anet authors that aligns raw ionic current events to their corresponding kmer positions in a reference, for the RNA004 data. This produces a per-read, per-position output table containing signal level features such as ionic current median, dwell time, standard deviation, and others. m6anet uses these features to predict the probability of an m^6^A modification occurring at a candidate DRACH position. We ran m6anet dataprep using the default maximum coverage of 1000 reads per transcript. None of the synthetic oligonucleotides achieved the m6anet 0.9 threshold for probability modified recommended for identifying a modified site. The synthetic construct m^6^A_1 was the highest with a score of 0.8986.

Additionally, we calculated performance metrics for Dorado performance on the synthetic oligonucleotides (Table 3). The Dorado Ψ model achieved both accuracy and F1-score in the range of 94% to 99%, while the Dorado m^6^A model showed accuracy between 94 and 98% and an F1-score ranging from 94 to 99%. Threshold-independent evaluation showed AUROC values ranging from 0.91 to 0.97 for Ψ and 0.98 to 0.99 for m6A, with AUPRC values of 0.96 to 0.99 for Ψ and 0.99 to 1.00 for m6A (Additional file 1: Fig. S5).

**Fig. 4:**
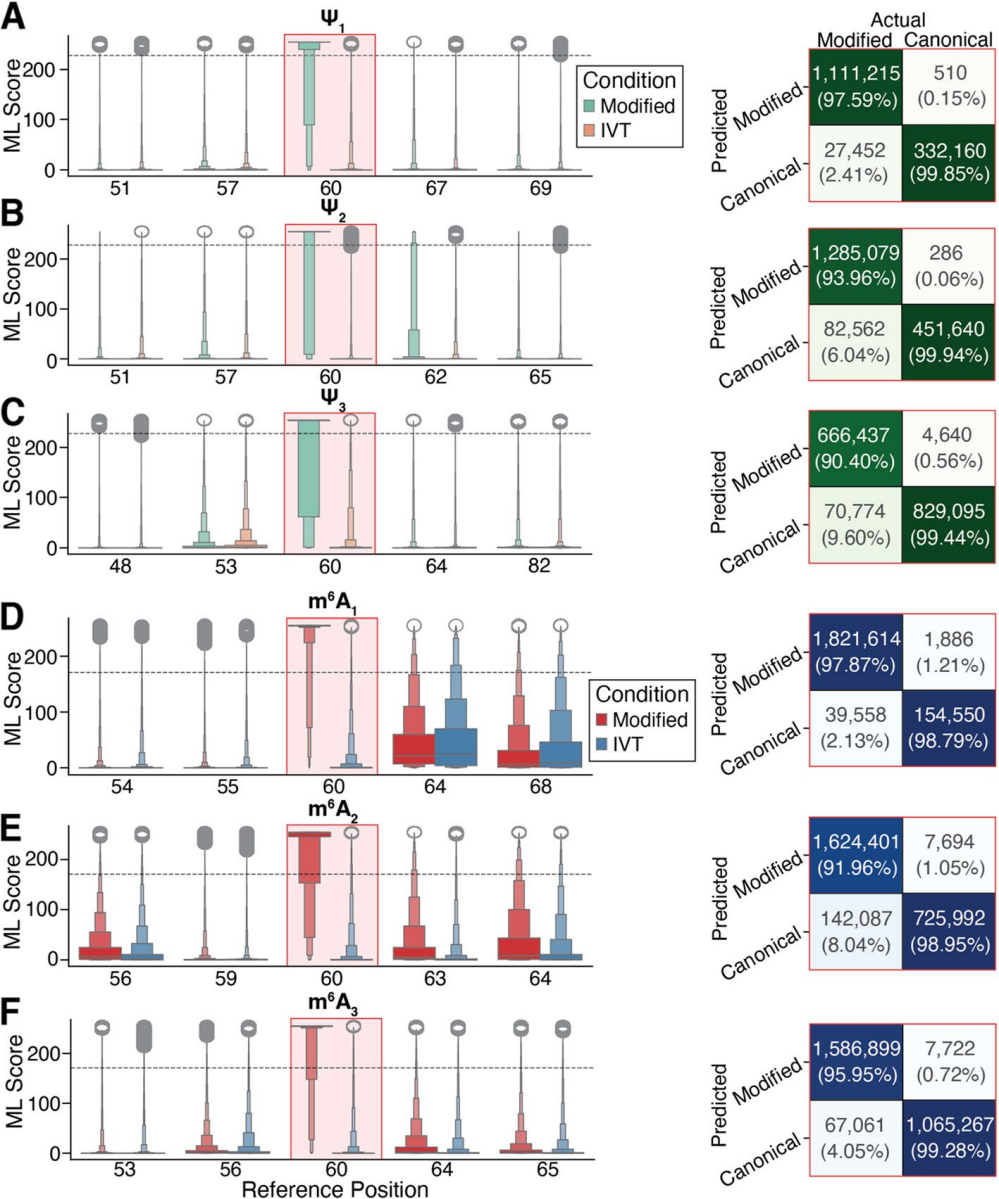
Evaluation of Dorado modification calling performance on known synthetic modification-bearing and canonical IVT oligonucleotides. Boxen plot comparing the raw Dorado modification likelihood (ML) scores at the known central modified site (60) and 2 reference U sites surrounding it for **A** Ψ_1, **B** Ψ_2, **C** Ψ_3, with confusion matrices of prediction at the modified site in each respective oligonucleotide. Boxen plot comparing the raw ML tag scores at the known central modified site (60) and 2 reference A sites surrounding it for **D** m^6^A_1, **E** m^6^A_2, **F** m^6^A_3, with a confusion matrix of prediction at the modified site in each respective oligonucleotide. The dashed gray line indicates the ML score threshold used for classification of modified versus canonical base. This threshold is determined using ONT's Modkit sample-probs function based on the 10th percentile of the ML score distribution

We analyzed the modification caller’s confidence at a per-read per-site basis for each of the oligonucleotides. We compared the modification-bearing oligonucleotide with the IVT oligonucleotide for both Dorado Ψ and m^6^A, at the known modified position and two reference bases surrounding that position (Fig. 4A-C and D-F).

We evaluated Dorado performance on these known modified conditions and their IVT pairs (Fig. 4A-C and D-F). True positives are where the modified base was correctly identified as modified (left upper box of the confusion matrix). False positives are where the canonical base is identified as modified (right upper box). False negatives are where the modified base was incorrectly identified as canonical (left lower box of the confusion matrix). True negatives are where the canonical base was correctly identified as canonical (right lower box of the confusion matrix). Dorado identified 90% to 98% of true positives, with less than 1% false positives across the six oligonucleotide pairs.

### Co-occurrence of multiple RNA modifications

**Fig. 5:**
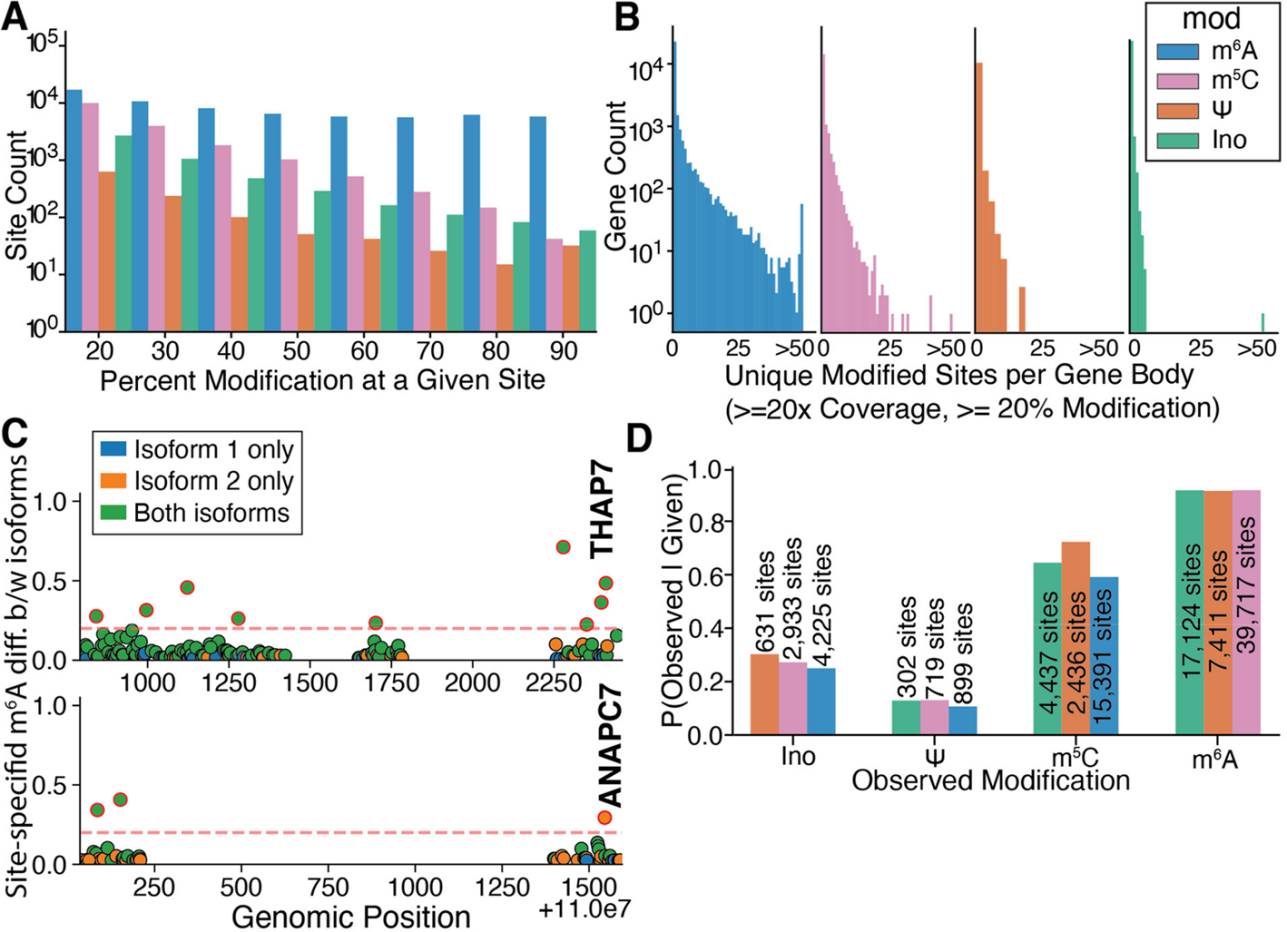
Dorado modification calling for Ψ, m^6^A, m^5^C and inosine in HEK293T.** A** Count of sites with ≥ 20 reads, and > 20% modification occupancy after subtracting 9-mer specific false positive rates. Counts per modification type were binned into 10% estimated modification occupancy windows starting at 20–30% occupancy and increasing by 10% increments. Each site is only counted once in this analysis in the exact bucket it belongs to. **B** Counts of GENCODE v47 genes binned by the number of occurrences of each modification across the entire gene body. Sites identified in subpanel A were aligned to GENCODE v47, and a pileup was generated for each gene to quantify the number of times a given modification was observed. The resulting counts represent the total modification occurrences per gene. **C** Selection of two exemplar genes with isoform-specific m6A expression. Each dot on the figure represents a common site between at least two isoforms of the given gene. The x-axis denotes genomic position, while the y-axis represents the maximal m^6^A percent occupancy distance between any two isoforms of that gene at that position. Positions that had a delta of greater than or equal to 20 are circled in red. **D** Co-occurrence of RNA modifications outside of a 5-nucleotide window in either direction, x-axis represents the observed modification, the y-axis represents the conditional probability of seeing that observed mod co-occurring with any of the three remaining given mods. When observing m^6^A, given m^5^C, Ψ, and inosine, there were 4,395, 791, and 1,857 genes with co-occurence. For m^5^C, given m^6^A, Ψ, and inosine, there were 4,395, 626, and 1,307 genes with co-occurence. For Ψ, given m^6^A, m^5^C, and inosine, there were 791, 626, and 262 genes with co-occurence. For inosine, given m^6^A, m^5^C, and Ψ, there were 1,857, 1,307, and 631 genes with co-occurence. The number of sites on these genes is listed on each respective bar

HEK293 has been used extensively to profile modifications using chemical-based next generation sequencing (NGS) methods. To draw comparisons to these orthogonal datasets, we sequenced HEK293 poly(A) RNA in three replicates (see “Methods”). The three experiments achieved 16,164,651 primary aligned reads from 22,515,770 (71.79%) total reads. Dorado basecalling was used to identify Ψ, m^6^A, m^5^C inosine, and 2'-O-Methyl modifications on each canonical base. Dorado called a total of 95,735 putative sites for Ψ, m^6^A, m^5^C and inosine, with ≥ 20 reads and ≥ 20% IVT-adjusted modification occupancy (Table 4). The number of modifications called when filtered for ≥ 10 reads and ≥ 10% IVT-adjusted modification occupancy are in Additional file 2: Table S2, and numbers for 2′-O-Methyl calls are in Additional file 2: Table S3. All four modifications showed a decreasing number of sites as occupancy cutoffs increased, with m^6^A showing similar numbers of sites for 60–70%, 70–80%, 80–90% and 90% + cutoffs. (Fig. 5A). We note that m^6^A had 5,801 sites with a reported occupancy of 90–100% (false-positive corrected). In contrast, Inosine, Ψ and m5c had 59, 32 and 42 sites with a reported occupancy of 90–100%, respectively. Modification calls for the comparative calls in GM12878 sequencing data can be found in Additional file 2: Table S4.

**Table 4:** Number of Dorado modification sites before and after subtraction of IVT false positive rates in HEK293

Mod	Number of sites before subtracting IVT (> 0%, > 0)	Number of sites before subtracting IVT (> 0%, > = 20 × coverage)	Number of sites before subtracting IVT (> = 20%, > = 20 × coverage)	Number of sites after subtracting IVT (> = 20%, > = 20 × coverage)
Ψ	1,284,252	1,018,223	3,707	3,103
m^6^A	3,486,693	2,459,262	70,058	67,517
m^5^C	2,411,357	1,706,439	26,772	18,159
Inosine	1,910,218	1,373,083	8,188	6,956

To investigate the concordance of DRS among three replicates of HEK293, we downsampled each of our datasets to the lowest number of reads present in any single dataset (~ 2.9 million). After calling mods with Modkit, we filtered modification sites for ≥ 20 read coverage in all three replicates, subtracted our IVT 9mer false positive rate from each site's reported modification percent, and filtered this IVT-adjusted modification occupancy ≥ 20% (Additional file 1: Fig. S6). For every modification we calculated the percentage of each super set as covered by any of the individual sets. This is known as Jaccard index, and results in 68% for m^6^A, 76.2% for Psi, 21.9% for Ino and 27.2% for m^5^C. To assess the quantitative reproducibility of Dorado modification calling across HEK293T replicates, we calculated the ICC(2,1) absolute agreement for each modification type, which evaluates whether each independent run of Dorado produces the same modification percentages at a given site. Replicates were aligned by site and filtered for sites where at least one replicate had ≥ 20% IVT-adjusted modification occupancy (Additional file 2: Table S5).

To better understand the distribution of modifications at a more granular level, we collated modification sites with at least 20 reads and 20% occupancy for each gencode v47 gene. We documented 5,848 genes with at least one modification (Fig. 5B), with 4,800 genes harboring more than one. We plotted the number of modifications passing our filtering criteria against their respective read lengths and observed a linear increase in modifications with read length (Additional file 1: Fig. S7). We next tested if the dorado models could detect isoform-level differences in modification profiles. Using Isoquant [26] and Modkit. We documented isoform-specific occupancy differences as high as 70% for m^6^A (THAP7), with other examples ranging from 20 to 55% (Fig. 5C).

Direct RNA sequencing is a unified technique to measure multiple modifications presenting the opportunity to measure co-observation of modifications at the gene level from a single sample. Here, we defined the co-observation rate as the proportion of genes with a modification B, given modification A on the gene body. We calculated the co-observation rate for all pairwise combinations of Ψ, m^6^A, m^5^C and inosine (Fig. 5D). To minimize the impact of neighboring modifications we only considered putative modification sites that were at least 5 bases away from the next closest putative modification site. The distance of 5 nucleotides was selected to center the putative modification site in a 9 nucleotide context window, the length of kmers in ONT RNA004 pore model [27], with no other modifications present. The co-observation rate for Ψ, the least frequent modification of our set, given any of the other three modifications, was at least 10.64%. Between Inosine, m^5^C, and m^6^A, the lowest co-observation rate was 24.98%. For genes containing at least one m^6^A call, co-occurrence ranged from 91.34% to 91.64% (Fig. 5D). Performing the same analysis on the GM12878 data revealed similar patterns. (Additional file 1: Fig. S8).

We also examined Dorado calls for all eight modifications in the GM12878 DRS data. For Ψ, m^6^A, m^5^C and inosine, Dorado called 144,609 putative sites (with valid coverage and ≥ 20% occupancy). We identified 119,804 high-confidence sites that each had 20 aligned reads with a modification occupancy of 20% or higher when adjusted for the 9-mer specific false positive rates. We also examined the Dorado calls for the four 2′-O-Methyl modifications (Additional file 2: Table S4, see “Methods”).

### Comparison of dorado modification calls with orthogonal datasets

**Fig. 6:**
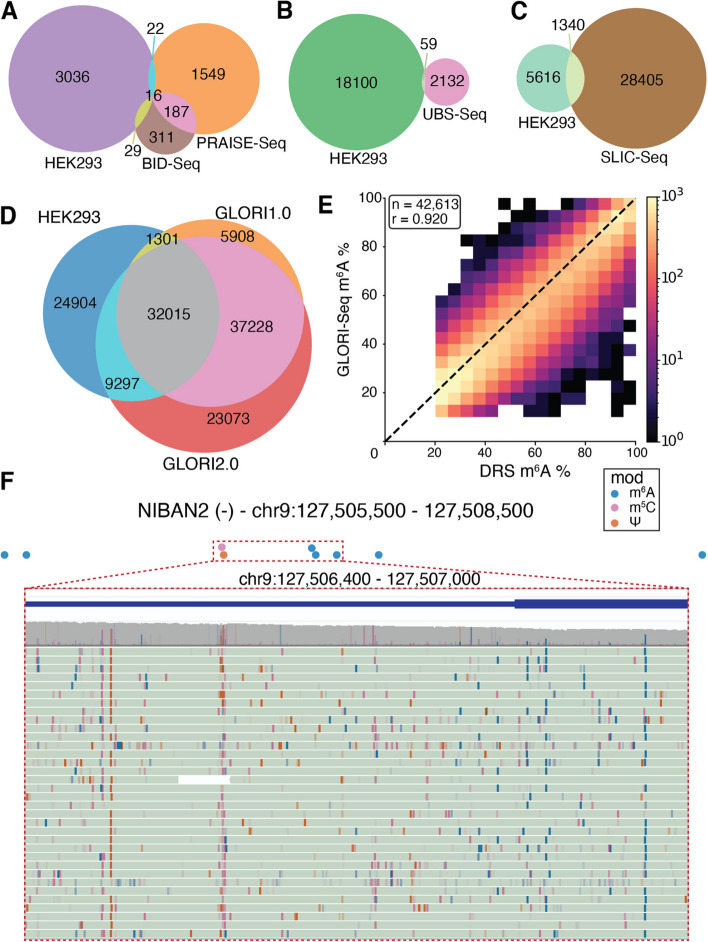
Comparison of Dorado modification calling to orthogonal methods. **A** Dorado Ψ modification calling in HEK293 when filtered for 20 reads in coverage and 20% in IVT-adjusted modification percentage, versus BID-Seq and PRAISE-Seq (HEK293) data specific to mRNA. **B **Dorado m^5^C modification calling when filtered for 20 reads in coverage and 20% in IVT-adjusted modification percentage in HEK293 vs. UBS-Seq (HEK293), filtered for only sites that occur in mRNA. **C** Dorado inosine filtered for 20 reads in coverage and 20% IVT-adjusted modification percentage calling in HEK293 versus SLIC-Seq (HEK293), filtered for mRNA sites. **D** Dorado m^6^A calling filtered for 20 reads in coverage and 20% IVT-adjusted modification percentage in HEK293 vs. GLORI-Seq 1.0 and GLORI-Seq 2.0 chemistries run on 10 ng of total RNA (HEK293). **E** Dorado m^6^A modification occupancy vs. the union of both GLORI-Seq modification sets, comparing modification occupancy at shared sites. **F** Exemplar gene NIBAN2, displaying sites for m^6^A, m^5^C, and pseudouridine that are all in their respective orthogonal datasets and within our Dorado HEK293 data when each modification was filtered for 20 reads in coverage and 20% IVT-adjusted modification occupancy

We identified orthogonal NGS datasets that used chemical alterations to document RNA modifications for HEK293 cells. Each orthogonal dataset was chosen based on the same cell line, HEK293T, to prevent any cell-line-specific modification differences. For this analysis, sites detected by DRS sites were filtered using the 20 reads and 20% IVT-adjusted modification occupancy thresholds. The orthogonal datasets were not filtered for sequencing depth or modification occupancy. For each comparison, we calculated the total number of unique sites (set intersection) and compared it to the number shared sites (set union), the proportion of shared sites over total sites is the Jaccard index. For Ψ, we used a BID-Seq dataset and a PRAISE-Seq dataset [28–31]. Dorado shared 29 of 543 sites with BID-Seq and 22 of 1744 sites with PRAISE-seq. The three techniques shared 16 total sites (Fig. 6A). BID-Seq and PRAISE-seq shared 203 out of 2,063 sites. For m^5^C, UBS-Seq [32, 33] shared 59 out of 2,191 sites with Dorado (Fig. 6B). Inosine profiling from SLIC-Seq [34, 35] shared 1,340 sites out of 29,745 with DRS (Fig. 6C). We used data from two different versions of GLORI-Seq chemistry, GLORI1.0, and GLORI2.0 [36, 37], that had been compared previously [36]. Dorado m^6^A calls agreed with the GLORI1.0 technique at 33,316 sites out of 76,452 GLORI1.0 sites, with a Jaccard index of 30.11% (Fig. 6D). For the GLORI2.0 technique, Dorado agreed with 41,312 sites out of 101,613 GLORI2.0 sites, with a Jaccard index of 32.32%. The GLORI1.0 and GLORI2.0 datasets had a Jaccard index of 63.63%. It should be noted that two HEK293T GLORI1.0 [38] datasets from separate publications had a Jaccard index of 37.46% (Additional file 1: Fig. S9).

GLORI-Seq also reports modification occupancy at each site it calls. Dorado shares this capability, making the direct comparison of modification proportion possible. We compared the occupancy from the union of the two GLORI-Seq datasets with Dorado m^6^A IVT-adjusted modification occupancy (Fig. 6E). For sites shared by the GLORI-Seq datasets we used the average of the two proportions. We found a high correlation between these two methods (r = 0.920). UBS-Seq, SLIC-Seq, and BID-Seq also provide modification occupancy measurements; the correlations and RMSE between all four DRS modifications and their orthogonal counterpart can be found in Additional file 2: Table S6. For 2′-O-Methyl modifications, we used NJU-Seq [39, 40] for comparison for each base. Dorado showed little to no agreement with these data (Additional file 1: Fig. S10).

We took the set of orthogonally validated HEK293 modification sites, meaning sites where both Dorado and all of the relevant orthogonal datasets identified a modified position, and intersected it with GENCODE v47. Based on these sites we were able to identify *GPR137* and *NIBAN2* as having m^6^A, m^5^C, and Ψ; and *ICMT* and *RPL37A* as having m^6^A, Ψ and inosine (Additional file 2: Table S7). An identical table was compiled for GM12878 (Additional file 2: Table S8), *ICMT and* RPL37A were shared between the two cell lines (Additional file 2: Table S9). Visual examination of the *NIBAN2* gene exemplifies the complexity of modification profiles on a single gene (Fig. 6F). This analysis was performed on the GM12878 sample as well. While the specific genes with multiple orthogonal modifications are different, the patterns seen in the comparison of HEK293 are similar (Additional file 1: Fig. S11).

This example demonstrates the advantages of simultaneous detection of multiple modifications at a single-molecule level. While the orthogonal methods can achieve single-molecule resolution, they typically only profile one modification at a time. LC–MS/MS can profile a large number of RNA modifications at once, but digest the mRNA into bases or fragments of RNA that cannot profile at the per-read level. Currently, Nanopore direct RNA sequencing is the only technology capable of simultaneously profiling multiple modifications on a single molecule.

## Discussion

In 2024, ONT released the new DRS RNA004 platform that had higher yields and improved accuracy compared to the older DRS RNA002 platform. This update also included a new basecaller (Dorado) that supported de novo detection of eight RNA modifications [17]: Ψ, m^6^A, m^5^C, Inosine and four 2′-O-Methyl nucleotides. Dorado enabled site-level modification analysis without requiring negative controls. We analyzed DRS data for poly(A) RNA from GM12878 B-lymphocyte cell line using both RNA002 and RNA004 platforms, and examined the eight RNA modifications that were detected by Dorado.

We compared orthogonal modification identification methods for Ψ (U-to-C mismatches) and m^6^A (m6anet). For both datasets, we observed site-specific differences in U-to-C mismatch and m^6^A occupancy despite originating from the same RNA sample. Traditionally, U-to-C mismatches were used as a method for detecting Ψ, but we observed significant discrepancies between U-to-C mismatch ratios and the Dorado Ψ modification caller. In contrast, we documented concordance for m^6^A calls between RNA002 (m6anet) and RNA004 (Dorado). This was understandable because both m6anet and Dorado use ionic current data to predict modifications.

We also evaluated Dorado modification calling performance using six synthetic oligonucleotides with known modifications (three for Ψ, three for m^6^A) alongside canonical IVT RNA controls. Dorado identified Ψ and m^6^A with an accuracy of 0.94–0.98, and an F1 score of 0.96–0.99. The false positive rate was around ~ 1%. The U-to-C mismatch percentage for the same known Ψ sites in these synthetic oligonucleotides varied between 2 and 22%.

While Dorado outperformed other modification detection strategies, it does report false positive calls. Thus, further validation and refinement is required to reduce Dorado-based false positives. These refinements include manually inspecting the modification calls (examining the sequence context and region), and considering a suitable coverage and modification occupancy threshold. To increase the specificity of Dorado calls, we adjusted their modification occupancy by subtracting false positive rates derived using genomic IVT DRS data.

We compared Dorado modification sites that met the filtering criteria across three technical replicates of HEK293, and documented concordance. We also investigated the co-occurrence of multiple modifications. We also compared DRS-based modification calls with orthogonal datasets for each of the eight modifications in HEK293 cell line, and observed discordance. However, we also documented discordance among orthogonal databases themselves. For example, our comparison revealed a ~ 63% agreement among existing m6A detection methods (GLORI1 and GLORI2). We also noted a ~ 37% similarity among different GLORI1 replicates. This suggests that while cross-validating DRS-based modification calls is essential, it is equally important and timely for the field to implement better orthogonal strategies that can curate “ground truth” modification datasets.

Finally, even within the limited subset of modification sites that agreed between DRS and orthogonal data, we identified candidate genes with suggestive evidence of multiple RNA modifications at a single-molecule level. The combination of single-molecule direct RNA analysis coupled with orthogonal comparison provides a unique lens to study RNA biology.

## Conclusions

The Nanopore DRS RNA004 platform has improved median accuracy (> 98.6% in this study) and can detect eight RNA modifications de novo including Ψ, m^6^A, m^5^C, inosine, and four 2′-O-Methy modifications. Comparing RNA modification detection methods between RNA002 and RNA004 DRS data revealed significant discrepancies. This suggests that methods originally developed for RNA002 DRS data may not directly translate to RNA004, especially the U-to-C mismatch-based strategies for pseudouridine identification.

We recommend the following steps for curating Nanopore DRS-based nucleotide modification calls: i) applying a minimum coverage threshold of 20 reads, ii) applying a minimum Dorado-based modification occupancy of 20%, iii) using canonical IVT DRS data for adjusting baseline false-positive rates in Dorado modification calls, and iv) validating modification calls using orthogonal methods including synthetic RNA and LC–MS. Additional caution should be exercised when investigating putative modifications that occur in low complexity regions or in close proximity to other putative modifications.

Our analyses further demonstrated that it is possible to detect multiple RNA modifications simultaneously, each at a per-read, per-site level, using the Nanopore DRS RNA004 platform. However, we argue that cross-validating modification calls using orthogonal techniques such as chemical assays, synthetic and biological controls, and LC–MS/MS will increase the confidence in any biological conclusions that are drawn using Nanopore DRS. With supporting orthogonal comparison, Nanopore DRS is a powerful tool for studying RNA modifications and their interplay in biological mechanisms and function.

## Methods

### GM12878 cell culture and RNA isolation

RNA002 poly(A) RNA data used in this study were from the 2019 study [1]. GM12878 cell culture, RNA extraction, and poly(A) RNA enrichment details have been previously described [1]. Briefly, low passage (passage 4) cells were cultured, and total RNA was isolated using Trizol-Chloroform extraction. This was followed by poly(A) RNA enrichment using NEXTflex Poly(A) Beads (BIOO Scientific cat. no. NOVA-512980) and stored at −80 °C. This poly(A) RNA was sequenced using RNA002 chemistry previously [1]. For this study, we rebasecalled the RNA002 dataset using Guppy v6.3.8 (hac model).

We extracted total RNA from GM12878 cells using the same protocol as previously described [1]. We used 2 μg of total RNA as input for the RNA004 experiment which was performed under standard conditions. These data were basecalled using Dorado [16] basecaller v1.0 with the super accuracy model (rna004_130bps_sup@v5.1.0).

dorado basecaller sup -–modified-bases pseU_2OmeU 2OmeG m5C_2OmeC

inosine_m6A_2OmeA

For both RNA002 and RNA004, reads were aligned to the human reference genome (hg38.p13) using minimap2 [41], then sorted and indexed using Samtools.

minimap2 -uf -k 14 -ax splice | samtools sort -o

samtools index

### HEK293 RNA isolation

We extracted total RNA from HEK293 cells using the same protocol as previously described [1]. We used 2 μg of total RNA per RNA004 experiments which were performed under standard conditions. We did three experiments for HEK293 using three PromethION flow cells. The data were processed using the same steps as we listed for GM12878.

### Biological modification calling

#### RNA002 U-to-C mismatch

To calculate U-to-C mismatches, we first ran Pysamstats [42] on RNA002 aligned, filtered, and sorted BAM file using the GRCh38 genome reference.

pysamstats –-type = variation_strand–- fields = "chrom,pos,ref,deletions,deletions_fwd,deletions_rev,insertions,insertions_fwd,insertions_rev,A,A_fwd,A_rev,C,C_fwd,C_rev,T,T_fwd,T_rev,G,G_fwd,G_rev"

To identify U-to-C mismatches, we first filtered for valid positions that have at least 20 reads. We analyzed positions where the reference base was T (representing U in RNA) and the observed base in sequencing reads was C. This calculation was repeated for the reverse strand. U-to-C mismatch was calculated as the number of C bases observed over the total number of bases at these positions. We limited our analysis to positions where this fraction was greater than zero (indicating at least one U-to-C mismatch). We then calculated the percentage contribution of each base (C, G, A) at these positions.

To ensure that U-to-C mismatches were not confounded by genomic variants, we used Clair3 [43], a variant caller designed for long-read sequencing, to exclude genomic variants from all analyses. We ran Clair3 on sequenced and aligned genomic DNA from GM12878 with the r1041_e82_400bps_sup_v500 model to identify positions with potential genomic variations. All variant positions were then excluded from the U-to-C mismatch calculations, eliminating genomic variation as a possible source of error. Ensuring that only genuine mismatches were considered in our analysis.

#### RNA004 Ψ modification calling and U-to-C mismatch

The same steps used for calculating U-to-C mismatches in the RNA002 dataset were applied to the RNA004 dataset. This included using Pysamstats, extracting positions where the reference base was T (U in RNA) and the observed base was C, computing the fraction of C reads filtering positions with at least one U-to-C mismatch, and calculating the percentage contribution of each base. This was repeated for the reverse strand. Additionally, genomic variants identified using Clair3 were excluded to ensure that only mismatches were considered.

For identifying putative Ψ sites in RNA004, the sorted and filtered BAM file originating from the Dorado Ψ modification caller was analyzed with ONT’s Modkit [44] tool to extract predicted modification information. We used modkit sample-probs to get the filter threshold based on the 10th percentiles of data.

modkit sample-probs –sampling-frac 0.1 –hist –seed 42

modkit pileup -–sampling-frac 0.1 –-filter-threshold T:0.90 -–filter-threshold C:0.80 –-filter-threshold A:0.65 -–filter-threshold G:0.87 -–seed 42

Only positions supported by at least 20 reads were considered in the final analysis of Modkit results. 

#### m6A Calling with m6anet and Dorado

To compare the RNA002 chemistry, we used m6anet [14] to detect m^6^A in DRS data. Reads were aligned to the Gencode v47 transcript [45] reference using Minimap2. The aligned BAM file was subsequently sorted, filtered to include only primary reads, and indexed using Samtools. Nanopolish [46] eventalign was used to produce event-level alignments for use with m6anet.

nanopolish eventalign -–scale-events -–signal-index

We used m6anet to identify putative m^6^a sites in the RNA002 data. m6anet’s HCT116_RNA002 model was used for inference. We called m^6^A sites in the RNA004 data using m6anet as well. The same series of steps was followed to generate these calls, with the exception of f5c [47] being used to produce the event-level alignments since nanopolish has not been updated for RNA004 data.

f5c eventalign –-rna –-pore rna004 -–signal-index –-scale-events -–min-mapq 0 -–min-recalib-events 10

Site-level information was tabulated using modkit pileup.

m^6^A calling was performed with both Dorado and m6anet. Once called, each dataset was filtered for a minimum coverage of 20 reads aligned at a given site. m6anet andModkit were called on the modified BAM files using:

 m6anet dataprep --eventalign

m6anet inference --pretrained_model HEK293T_RNA004 --num_iterations 20

modkit pileup --sampling-frac 0.1 --filter-threshold T:0.90 --filter-threshold C:0.80 --filter-threshold A:0.65 --filter-threshold G:0.87 --seed 42

#### Modification occupancy adjustment and re-filtering with whole genome IVT data

Using a 9-mer false positive rate table [23], we calculated the reported modification occupancy of each site in the modkit pileup. After subtracting the false positive rate, we refiltered for sites that continued to have a reported modification occupancy over 20% and a minimum of 20 valid (Modified or Canonical) read coverage.

python IVT_fp_correction.py \

--modkit input_bedMethyl.tsv \

--reference reference_genome.fa \

--errortable

GM12878_genomic_IVT_refmatch_9mer_all_threshold.dorado_1.0.tsv.gz

--outpath corrected_bedMethyl.tsv

--valid_only \

--filter_mismatch \

--filter_kmer

#### Selection of sites for designing synthetic oligonucleotides

To evaluate the performance of Dorado modification calling and the traditional method used for detecting Ψ and m^6^A on known modification states, we designed 6 synthetic oligonucleotides and their corresponding in vitro transcribed pairs.

#### Ψ site selection criteria

For the RNA002 dataset, we matched positions and chromosomes between Pysamstats U-to-C mismatch data and Dorado Ψ predictions. We applied filters requiring at least 20 reads and categorized positions into three groups:

- Ψ_1: High U-to-C mismatch & High Dorado Ψ (both 100%)
- Ψ_2: High U-to-C mismatch & Low Dorado Ψ (U-to-C = 100%, Dorado Ψ = 0%)
- Ψ_3: Low U-to-C mismatch & High Dorado Ψ (U-to-C = 0%, Dorado Ψ = 100%)

We then analyzed the resulting sequences, excluding positions located in homopolymer regions. The identified regions were visually inspected using the Integrative Genomics Viewer (IGV) [48], ensuring that no designed oligonucleotide spanned an exon junction. Further structural and thermodynamic analyses were performed, including secondary structure prediction using RNAfold and ΔG (Gibbs free energy) calculations to assess sequence stability, dimer formation, and homodimer probability analysis to minimize unwanted interactions, melting temperature, GC content, and other physicochemical properties analyzed using Integrated DNA Technologies (IDT) tools. The three sequences we selected met all these criteria.

#### m^6^A site selection criteria

We calculated site-specific occupancy differences between Modkit’s output and the genomic positions calculated from m6anet’s output. This allowed us to select a set of sites that represented large differences in m^6^A occupancy as calculated by the two technologies:

- m^6^A_1: High m6anet & Low Dorado (0th—5^th^ percentile of delta occupancy)
- m^6^A_2: Low m6anet & High Dorado (85th—95^th^ percentile of delta occupancy)
- m^6^A_3: High m6anet & High Dorado (> = 90% modified for both)

### Synthetic oligonucleotide design

Based on the selection criteria mentioned, we designed six 30-base-long sequences, three for Ψ and three for m^6^A. A known modification was placed in the middle of each sequence. To be able to sequence the selected synthetically modified oligonucleotides, we performed a splinted ligation strategy, attaching a 3′ and 5′ oligonucleotide (Additional file 1**: **Fig. S12, Additional file 2: Table S10). The 3′ oligonucleotides included a 3′ terminal sequence (polyadenylated tail) that made the full structure compatible with ONT’s DRS sequencing kit. The 3′ (60 bases) and 5′ oligonucleotide (46 bases) extended the total sequence length to 136 bases. A fully complementary single-stranded DNA template (ssDNA) was reverse-transcribed to hybridize with all oligonucleotides.

### Synthetic oligonucleotide experimental protocol

During the annealing process, RNA oligonucleotide, ssDNA, 3′ oligonucleotide, and 5′ oligonucleotide (1 μL of 100 μM each) were mixed with 6 μL of annealing buffer (10 mM Tris–HCl, pH 8.0, 50 mM NaCl) to a final volume of 10 μL at 10 μM concentration per tube. The mixture was then incubated at 75 °C for 2 min and reduced to 25 °C at a rate of 2 °C per minute before being held at 4 °C to facilitate annealing. For the ligation step, T4 RNA Ligase 2 (NEB #M0239) was used to ligate the adapter. The reaction mixture consisted of 5 μL of 10 μM of each construct (from the annealing step), 4 μL of 5X Quick Ligase Buffer (NEB # B6058), 8 μL of ultrapure water, and 3 μL of T4 RNA Ligase 2, bringing the total volume to 20 μL. The mixture was incubated at room temperature for 1 h. This was followed by a 1.5X SPRI beads (Beckman Coulter # B23318) clean up with 2 wash steps using 70% ethanol.

We used IVT to synthesize a true negative control to compare to the synthetic oligonucleotides. We designed 6 DNA oligonucleotides that contained the reverse complement sequence to the synthetic oligonucleotides in the 5′ and modified sections (Additional file 2: Table S10). To differentiate the true positive modified and true negative IVT from a mixed sample, we added a 15-nucleotide barcode near the 3′ end of the IVT template oligonucleotide. We also included a T7 polymerase targeting site at the 5′ end of the DNA reverse complement oligonucleotide. The complementary strand was generated using a one-cycle PCR reaction. For each template, the reaction mixture included 1 μL of 10 mM DNA template, 1 μL of 10 mM dT Primer, 12.5 2 × Long AMP Taq (NEB #M0323), and 10.5 μL of RNase-free water, which were combined in a separate tube for each template. Using the HiScribe NEB T7 High Yield RNA synthesis kit (#E2040), we transcribed true negative controls for our modified oligonucleotides. After in vitro transcription, reactions were treated with DNase I (NEB #M03030) for 15 min. Concentrations of all RNA oligonucleotides were determined with NanoDrop.

Following the DRS library preparation protocol with SQK-RNA004 kit [49], the libraries for 12 oligonucleotides were made in a single tube (a total of 9.5 uL RNA with 32 ng of each construct). The RNA RC, RNaseOUT, and the optional reverse transcription step were skipped and the protocol was followed through sequencing. The library was sequenced using an RNA004 flow cell on a PromethION platform for 72 h. The resulting Pod5 files were basecalled with Dorado 0.8.1 with modification calling enabled for Ψ and m^6^A separately.

dorado basecaller sup -–modified-bases pseU_2OmeU 2OmeG m5C_2OmeC inosine_m6A_2OmeA -–modified-bases-threshold 0 –-no-trim –-emit-moves

We used the complete sequence of all 12 oligonucleotides to create the reference. This reference was used with BWA-MEM for alignment.

bwa mem -W 13 -k 6

The resulting Sam file was converted to Bam, sorted and indexed using Samtools. Then, we copied the modification tags (MM and ML) from the Dorado unaligned BAM to the bwa-mem aligned BAM. Using the barcode attached to the end of the IVT oligonucleotides, we filtered the data to only include reads that aligned between the 5′ end of the synthetic 30-nucleotide sequence and the 3′-end of the barcode. This process was performed as well at positions that correspond to those indices in the synthetic modified oligonucleotides.

### Statistical analysis

Per read identity was calculated as the number of matches divided by the number of matches + mismatches + insertions + deletions for reads aligned to gencode.v47. We additionally limited identity calculations to reads with an aligned length of at least 200 (reference end—reference start).

To calculate the base substitutions, we processed sequencing data by reading the Pysamstats result. At each position, we extracted the reference base and the observed base counts (A, C, G, T). Positions where the base count was at least 20 were considered valid. For these valid positions, the reference base was compared to the observed bases. Then, we tracked how often each reference base was substituted by another base. Additionally, we computed the overall fraction of each base substitution by summing the base counts for each reference nucleotide.

To determine the number of genes observed in each of the datasets, we intersected each read's aligned coordinates with the gencode.v47 gene body coordinates. We did not restrict the number of overlapping nucleotides between the read and the gene body to be counted as an observation for a gene. A gene was considered observed in a dataset if there were at least 10 supporting reads.

### Poly(A) tail length calling

For RNA002 data, polyA tail lengths were estimated using nanopolish.

nanopolish polya

For RNA004 polyA, tail lengths were estimated using Dorado.

dorado basecaller -–estimate-poly(A)

We grouped aligned reads into mitochondrial-sourced transcripts and genomic transcripts based on the aligned chromosome for each read.

#### Analysing performance for synthetic oligonucleotides

To compare Dorado modification likelihood (ML) values for synthetic oligonucleotides and their IVT pair, we captured the read position, reference position, base call, and ML tags for each read. The ML tag is representative of the model’s confidence that a given base is modified. This allowed us to compare the model's performance at the known modified sites and two matching bases around it (uridine for Ψ or adenosine for m^6^A) in both the modified and IVT negative conditions.

To evaluate the performance of Dorado on modified and canonical bases, we made confusion matrices for the modified positions and IVT pairs. This shows a detailed summary of Dorado’s performance by comparing the predicted condition with the true condition. True Positives (TP): where the modified base was correctly identified as modified. True Negatives (TN): where the canonical base was correctly identified as canonical. False Positives (FP): where the canonical base was incorrectly identified as modified. False Negatives (FN): where the modified base was incorrectly identified as canonical. The summary statistics of model performance were computed (Table 1) using the following formulas:

$$Balanced\; Accuracy =\frac{{sensitivity}+{specificity}}{2}$$

$$Accuracy = \frac{TP + TN}{TP + TN + FP + FN}$$

$$Precision= \frac{TP}{TP + FP}$$

$$Specificity=\frac{TN}{TN + FP}$$

$$Recall (Sensitivity)=\frac{TP}{TP + FN}$$

$$F1\; score = \frac{2(Precision * Recall)}{Precision + Recall}$$

To assess threshold-independent classification performance, we generated Receiver Operating Characteristic (ROC) and Precision-Recall (PR) curves using per-read modification probabilities extracted via modkit extract. For each read at the target modified position, we derived the modification probability from the ML tag: reads called as canonical were assigned a modification probability of 1, call confidence, while reads called as modified retained their call confidence as the modification probability. Ground truth labels were assigned based on sample origin, where reads from modified samples were labeled as positive (modified) and reads from IVT samples were labeled as negative (canonical). The Area Under the ROC Curve (AUROC) and Area Under the Precision-Recall Curve (AUPRC) were computed to quantify overall discriminative performance across all classification thresholds.

#### Filtering criteria

To identify putative modification sites from the GM12878 and HEK293 data, we selected the filtering criteria of a minimum of 20 reads with reference-matching nucleotides aligned at the position (not mismatch, insertion, or deletion) with a minimum of 20% predicted modification occupancy. These criteria were selected based on the false positive rate observed using the synthetic oligonucleotide sequencing results. Assuming the highest false positive rate of 0.0121 as the success parameter for a binomial distribution, our filtering criteria give a probability of a false positive putative modification site as 0.0000854758 or less than 1 in 10,000 sites. We used this criterion to limit false-positive sites. We also tested 10 read coverage and 10% modification occupancy and found 20 read coverage and 20% occupancy to be more reliable.

To identify isoform-specific modifications, we used Isoquant [26], which produced a per-read isoform classification.

isoquant.py -–data_type nanopore

Using the Modkit extract calls feature intersected with the isoform classification, we made an isoform-specific modification pileup. Isoform positions were transformed to genomic positions and isoform-specific modification differences were calculated.

modkit extract calls

#### Orthogonal datasets

The orthogonal datasets used for comparison with our Dorado HEK293 DRS were sourced through a literature review. For the comparison of pseudouridine, PRAISE-Seq Supplementary Dataset 2 [29] and BID-Seq [29, 30]. PRAISE sites were filtered for any sites that were identified on ncRNA, leaving only mRNA sites remaining. For m^5^C, UBS-Seq, the wild-type mRNA identified sites in HEK293T were used from the Gene Expression Omnibus [32, 50]. These data were then filtered for sites that were called on mRNA, excluding sites called on tRNA and rRNA. For inosine SLIC-Seq was used for comparison [34]. These sites were filtered for only sites that occur in UTRs, intergenic, or exonic. For m^6^A, GLORI1.0 and GLORI2.0 chemistries performed on HEK293T were used for comparison [35, 36], as well as a previous publication of the GLORI1.0 chemistry [37]. For comparison of 2’Omethyl, NJU-Seq performed on HEK293T mRNA was used [38, 39].

## Supplementary Information

Additional file 1.

Additional file 2.

## Data Availability

All the scripts and codebase used in this study are publicly available with an MIT license in GitHub repository (https://github.com/genometechlab/RNA002_vs_RNA004) [51] and Zenodo (https://zenodo.org/records/19338093) [52]. Processed modkit data are available in Zenodo (https://zenodo.org/records/19338093) under accession number 19338093 [52]. GM12878 RNA002 sequence level data are publicly available from AWS (https://github.com/nanopore-wgs-consortium/NA12878/blob/master/RNA.md). GM12878and HEK293 RNA004 sequence level data are publicly available through the European Nucleotide Archive (https://www.ebi.ac.uk/ena/) under Project Accession PRJEB111137 [53]
