# Supplementary material for: Assessment of nanopore RNA modification calling in human cell lines and synthetic systems
Source: Genome Biol. 2026 May 7;27:190. doi: 10.1186/s13059-026-04096-w (PMC13251006; doi:10.1186/s13059-026-04096-w)
Supplement: Supplementary file 1 — Additional file 1. [file 13059_2026_4096_MOESM1_ESM.docx]

| 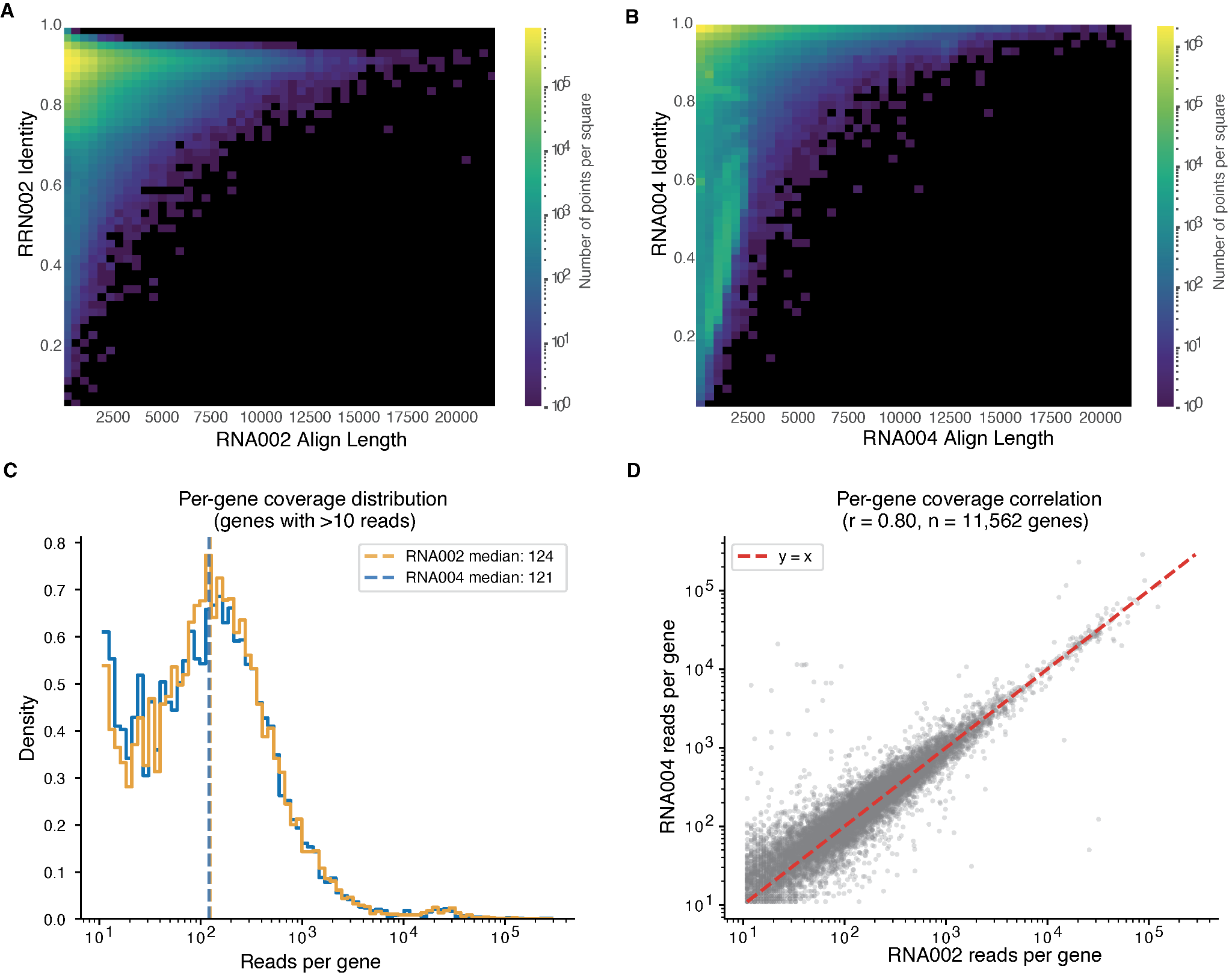 |
| --- |
| **Fig S1. A.** Align length vs. alignment identity for RNA002. **B**. Align length vs. alignment identity for RNA004. **C.** Per-gene coverage comparison between RNA002 and RNA004. Distribution of reads per gene for genes with >10 aligned reads. Dashed lines indicate median values. **D.** Correlation of per-gene read counts between chemistries (Pearson r = 0.80, n = 11,562 genes). |

| 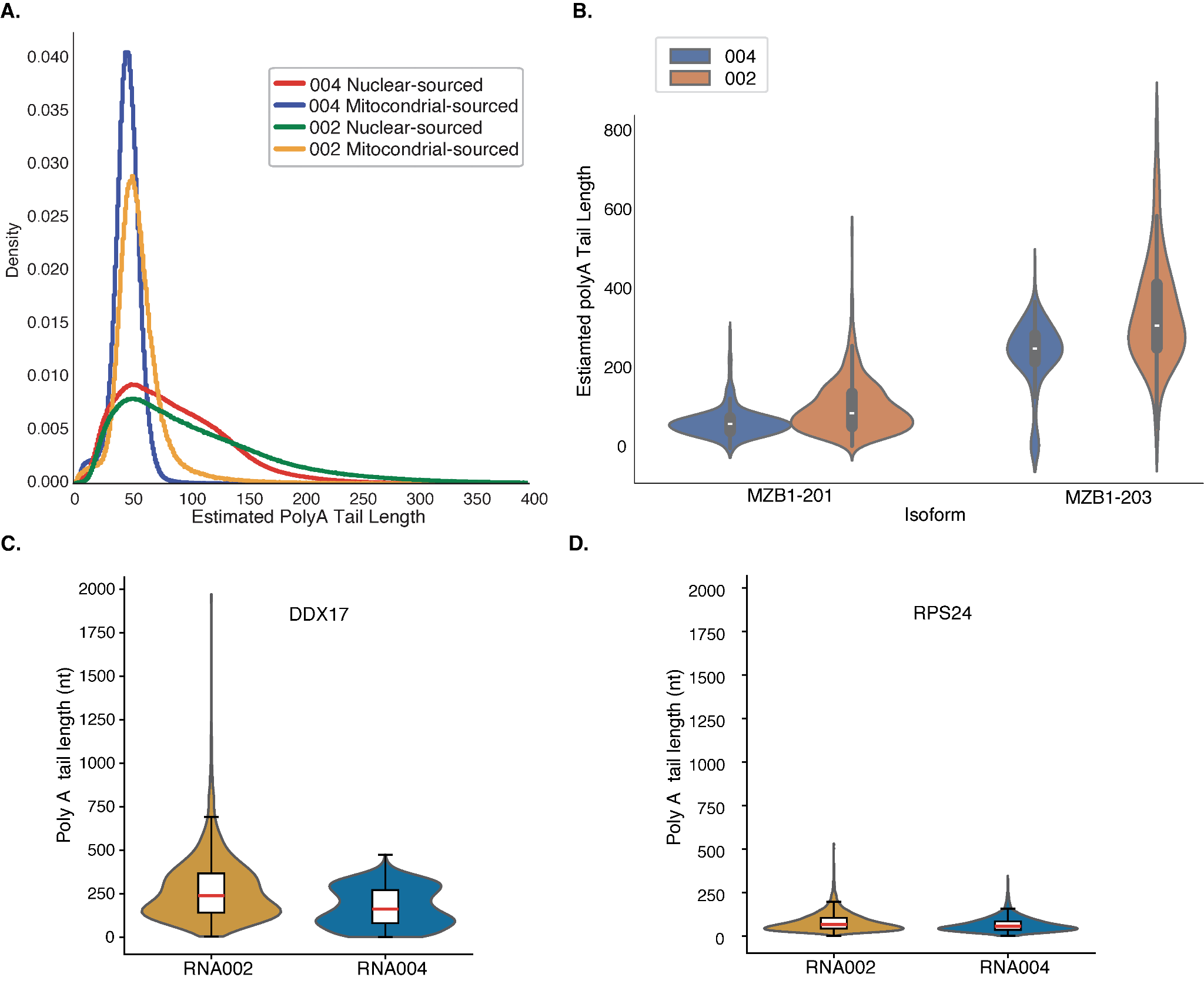 |
| --- |
| **Fig S2. A.** poly(A) tail estimates for mitochondrial and cytosolic transcripts for RNA002 (nanopolish) and RNA004 (Dorado). **B.** An exemplar gene (*MZB1*) with significant Isoform-specific differences in poly(A) tail estimation for RNA002 and RNA004. Estimates for RNA002 were produced with nanopolish polya, while RNA004 estimates were produced with Dorado. **C.** Violin and box plots showing polyA tail length distributions for *DDX17* (long polyA). **D.** Violin and box plots showing polyA tail length distributions for *RPS24* (short polyA). |

| 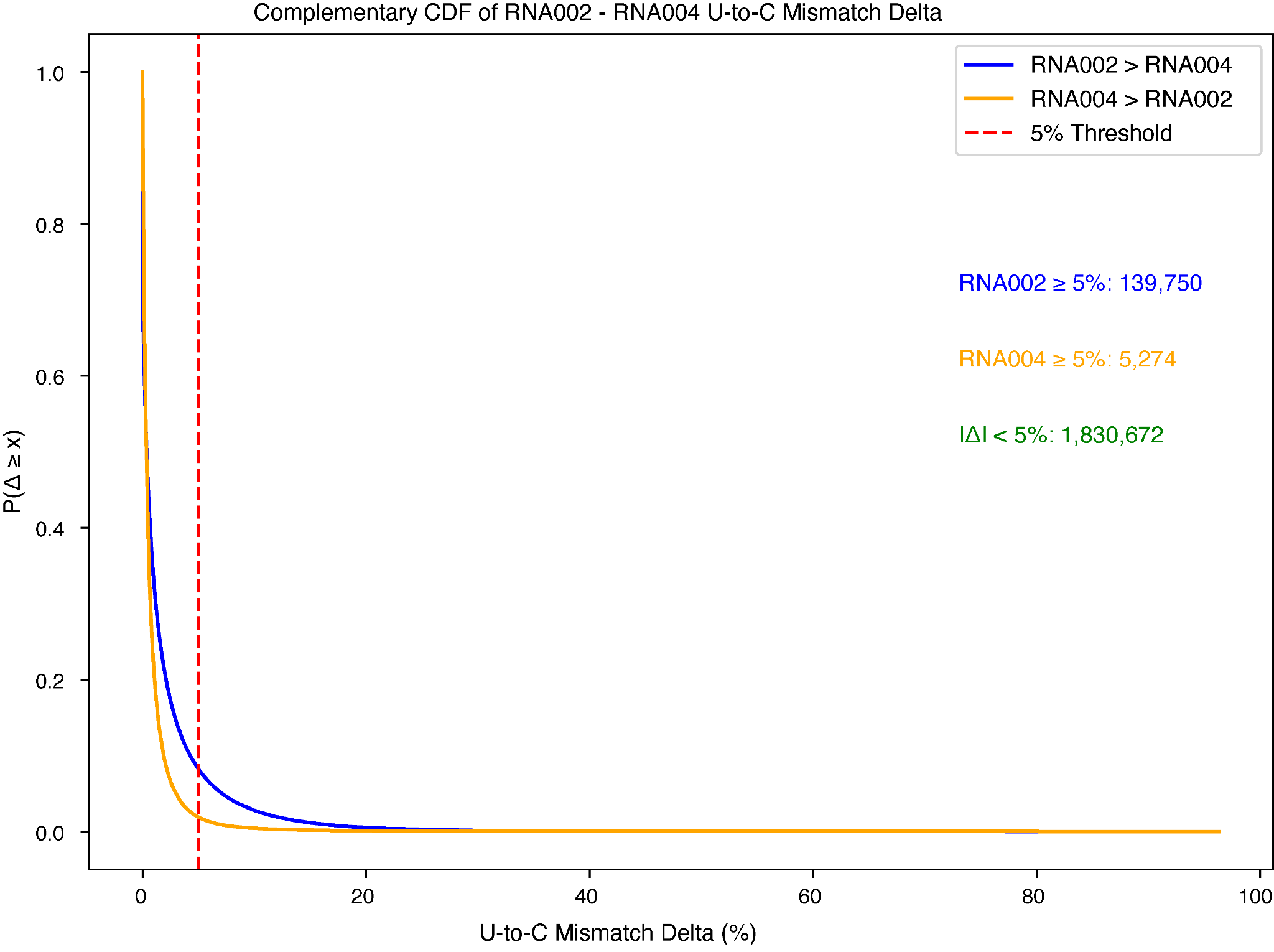 |
| --- |
| **Fig S3.** Complementary cumulative distribution plot of site-level U-to-C mismatch differences between RNA002 and RNA004. The x-axis represents the mismatch delta (RNA002 − RNA004) in percentage, and the y-axis shows the proportion of sites with a mismatch difference greater than or equal to 5%. Blue and orange lines correspond to sites where RNA002 or RNA004 had higher mismatch, respectively. The dashed red line marks the 5% delta threshold used to define comparisons. |

| 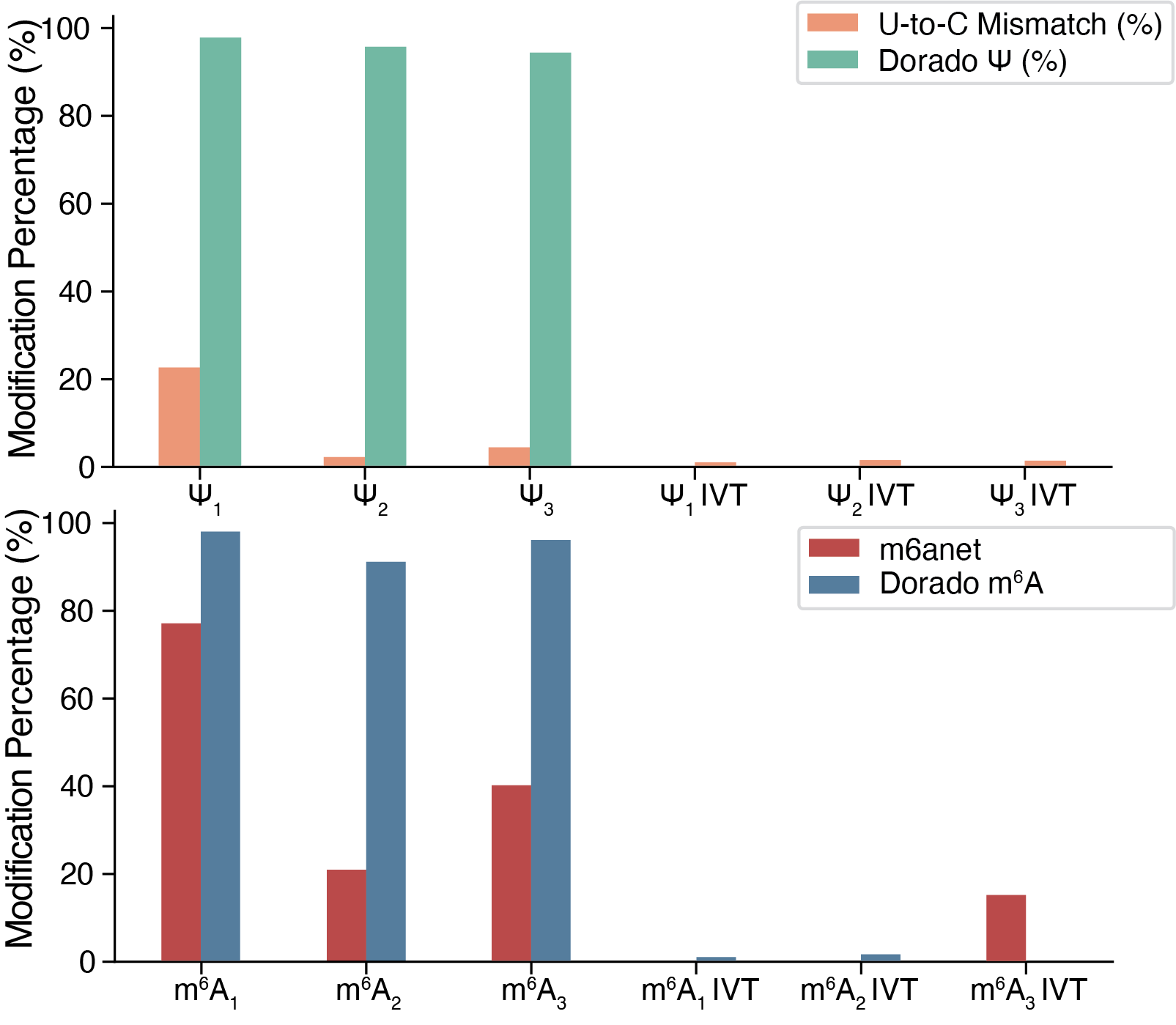 |
| --- |
| **Fig S4. A.** Reported modification occupancy percentage at the known modified site in synthetic oligos and IVT pair using both U-to-C mismatch and Dorado Ψ. **B.** Reported modification occupancy percentage at the known modified site in synthetic oligos and IVT pair using both m6Anet and Dorado m^6^A. |

| 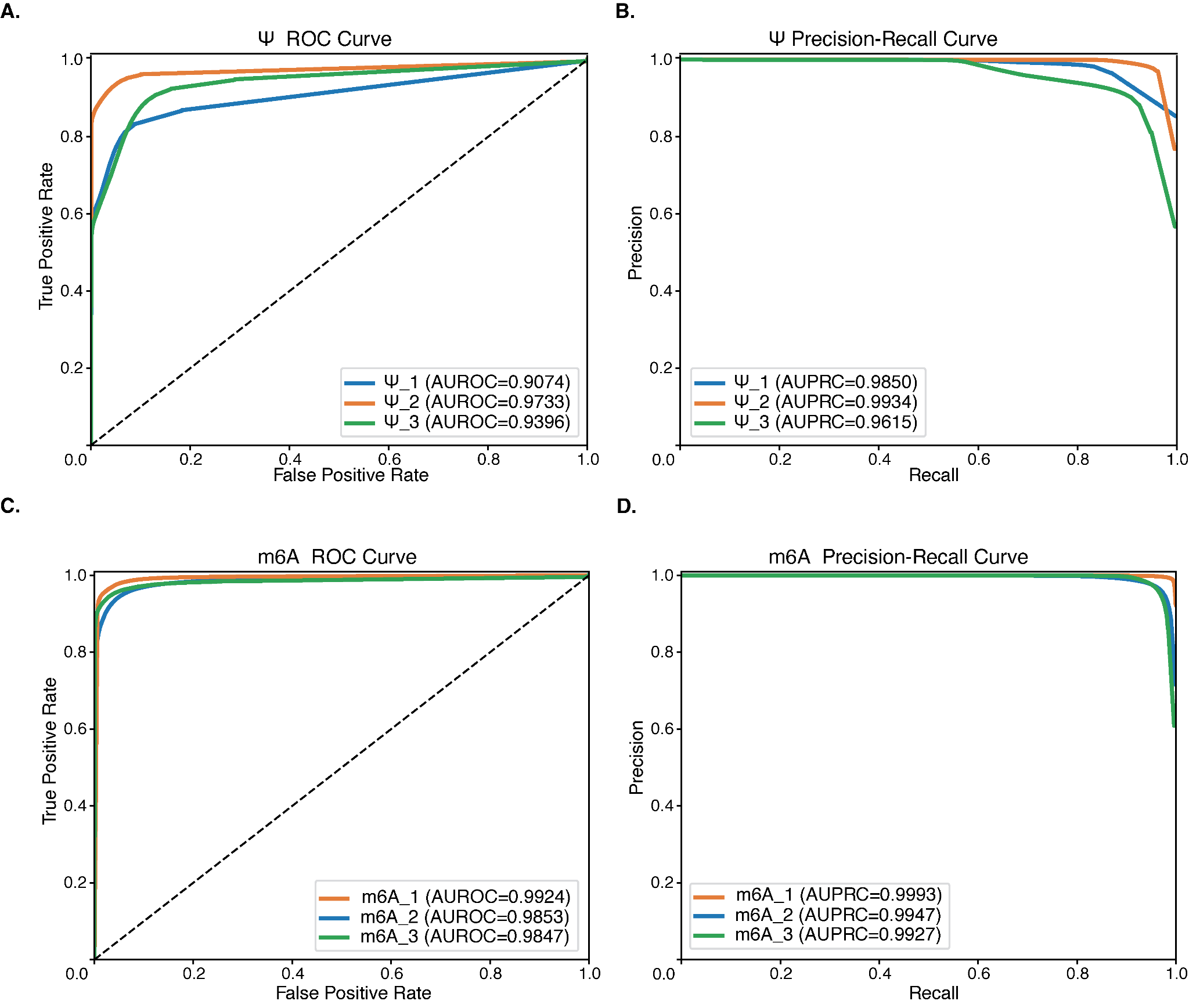 |
| --- |
| **Fig S5. ROC and Precision-Recall curves for Dorado modification detection on synthetic oligonucleotides. A.** ROC curves for Ψ detection across three synthetic oligonucleotide pairs. **B.** Precision-Recall curves for Ψ detection. **C.** ROC curves for m6A detection across three synthetic oligonucleotide pairs. **D.** Precision-Recall curves for m6A detection. AUROC and AUPRC values are shown in legends. |

| 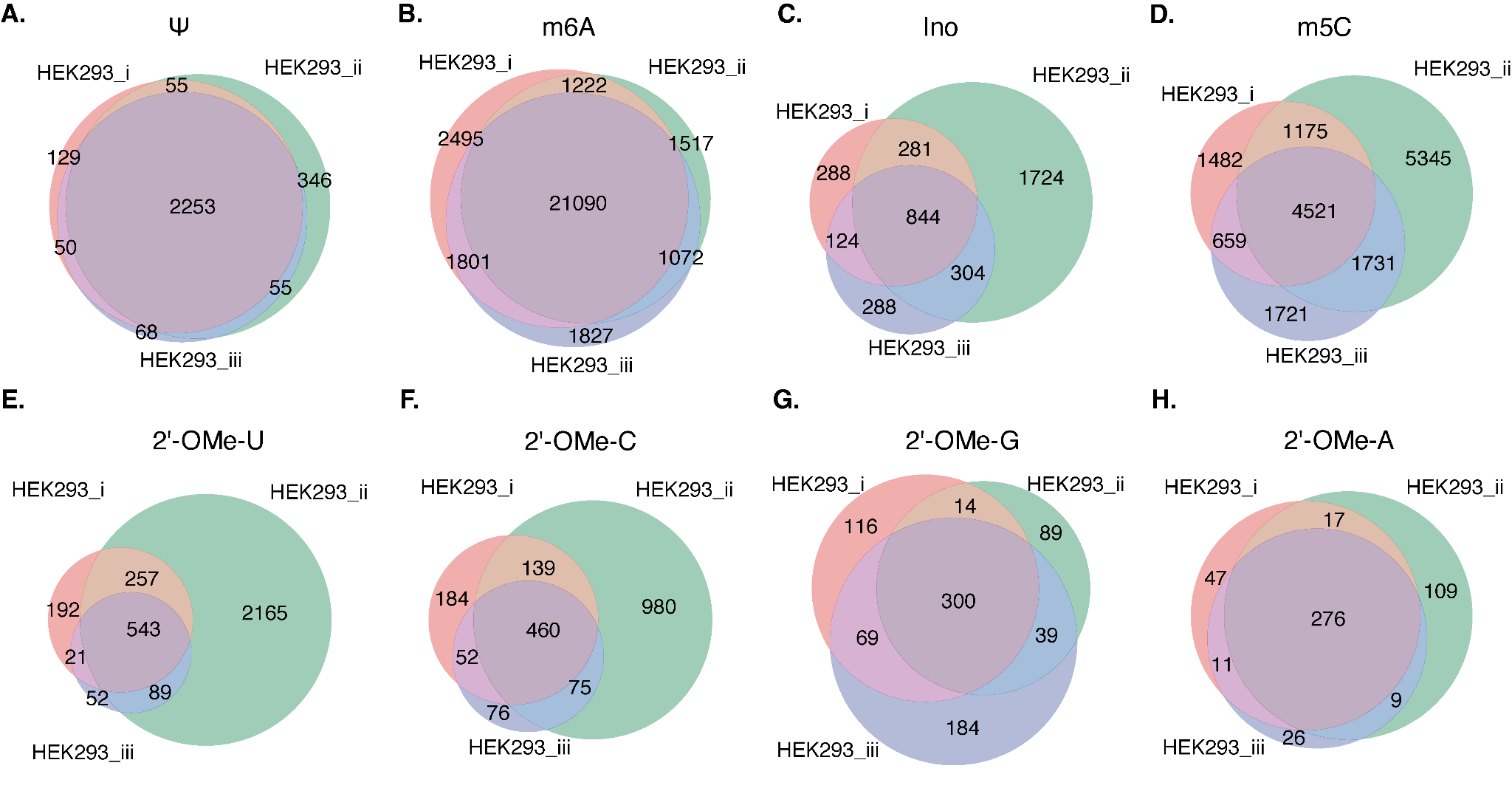 |
| --- |
| **Fig S6.** Three-way Venn diagrams comparing Dorado RNA modification sites across three biological replicates of HEK293 cells. Each replicate was subsampled to 2.9 million reads and analyzed using Dorado modification calling for eight modification types. Sites were filtered to include only those with ≥20 read coverage in all three replicates and IVT-adjusted modification occupancy ≥20%. Numbers indicate the count of unique modification sites in each region. **A.** Ψ sites with 2,253 sites shared across all three replicates. **B.** m6A sites with 21,090 shared sites (the most abundant modification detected). **C.** Inosine sites with 844 shared sites. **D.** m5C sites with 4,521 shared sites. **E.** 2'-O-methyluridine sites with 543 shared sites. **F.** 2'-O-methylcytidine sites with 460 shared sites. **G.** 2'-O-methylguanosine sites with 300 shared sites. **H.** 2'-O-methyladenosine sites with 276 shared sites (the least abundant modification). |

| 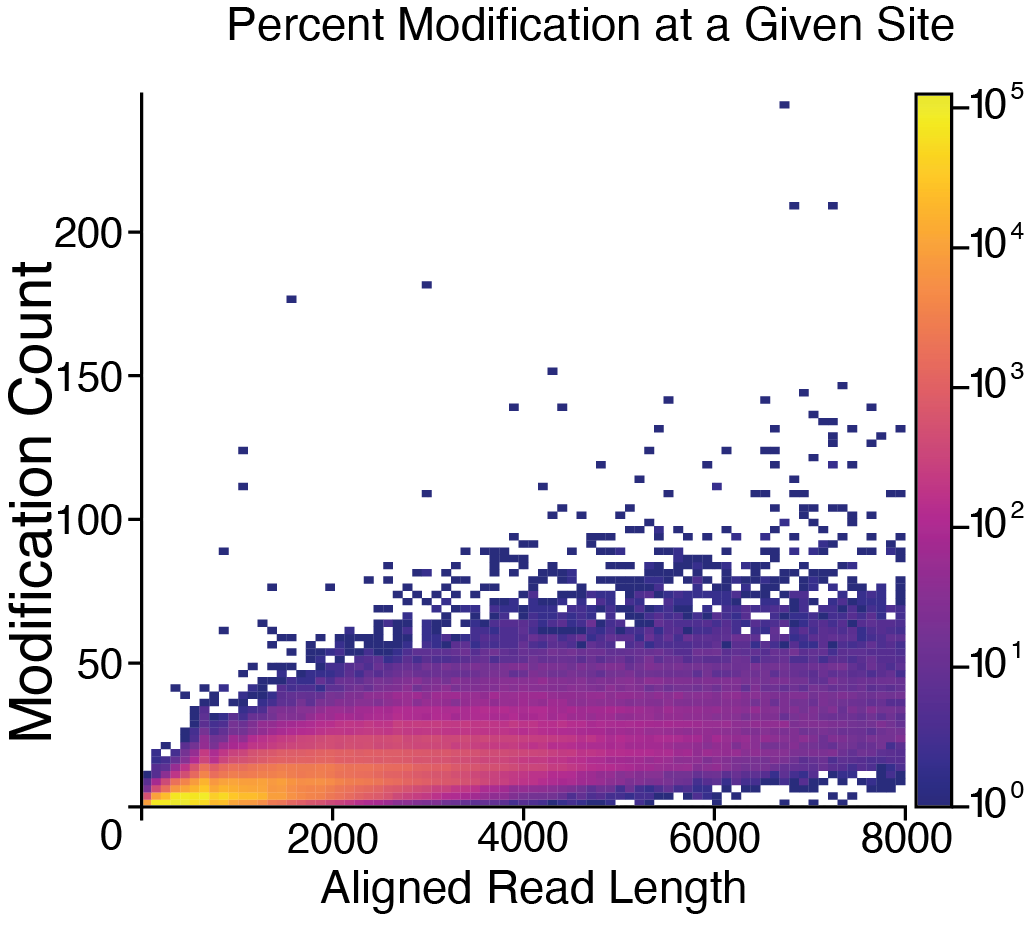 |
| --- |
| **Fig S7.** **Total number of valid modifications binned by aligned read length.** The x-axis represents that aligned read length for any read in our analysis, while the y-axis represents that total count of filtered modifications on that read. The colors in the heatmap represent the density of reads in a given bin, while the whitespace represents no reads in a given bucket. |

| 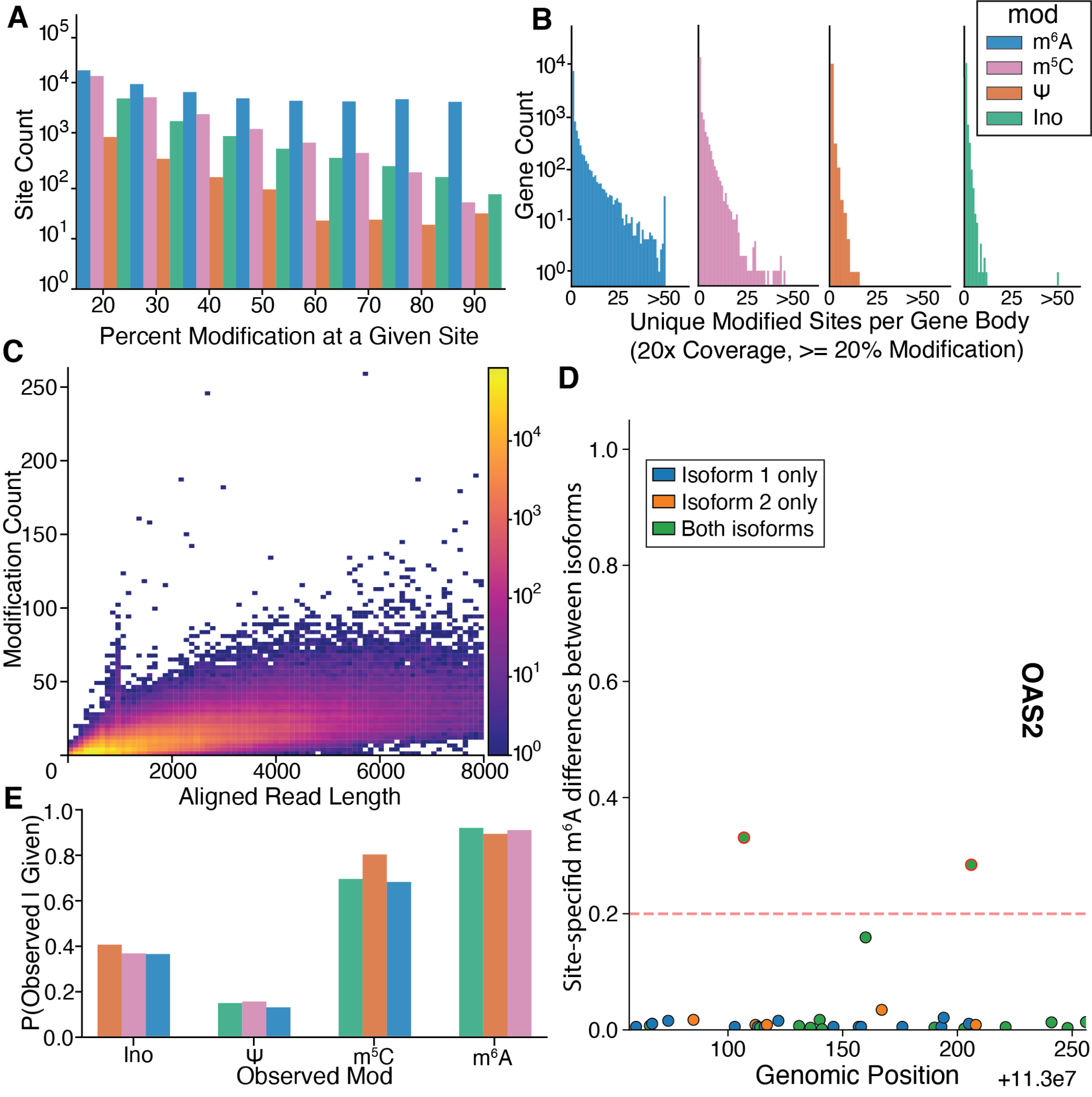 |
| --- |
| **Fig S8. Dorado modification calling for Ψ, m^6^A, m^5^C and inosine in GM12878 A.** Count of sites with 20 or more reads, 20% modification occupancy after subtracting 9-mer specific false positive rates. Counts per modification type were binned into 10% estimated modification occupancy windows starting at 20-30% occupancy and increasing by 10% increments. **B.** Counts of genes binned by the number of occurrences of the given modification across the entire gene body. Modifications were only counted if they met the same 20 read coverage with 20% corrected modification occupancy. **C.** Total number of valid modifications binned by aligned read length. The x-axis represents that aligned read length for any read in our analysis, while the y-axis represents that total count of filtered modifications on that read. The colors in the heatmap represent the density of reads in a given bin, while the whitespace represents no reads in a given bucket. **D.** Selection of three exemplar genes with isoform-specific m6A expression. Each dot on the figure represents a common site between at least two isoforms of the given gene. The x-axis denotes genomic position, while the y-axis represents the maximal m^6^A percent occupancy distance between any two isoforms of that gene at that position. Positions that had a delta of greater than or equal to 20 are circled in red. **E.** Co-occurrence of RNA modifications outside of a 5-nucleotide window in either direction, x-axis represents the observed modification, the y-axis represents the conditional probability of seeing that observed mod co-occurring with any of the three remaining given mods. |

| 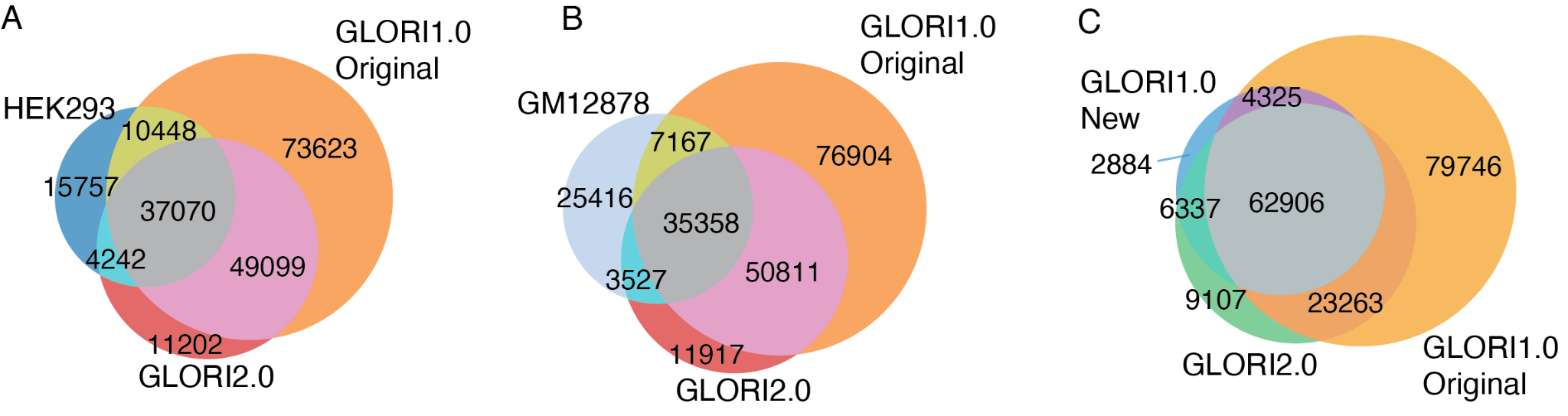 |
| --- |
| **Fig S9.** **Original GLORI1.0 Chemistry overlap with DRS and update samples.** **A.** HEK293T DRS overlap with original GLORI1.0 and GLORI2.0. **B.** GM12878 DRS overlap with original GLORI1.0 and GLORI2.0. **C.** Original GLORI1.0 chemistry overlap with updated GLORI1.0 and GLORI2.0 results. |

| 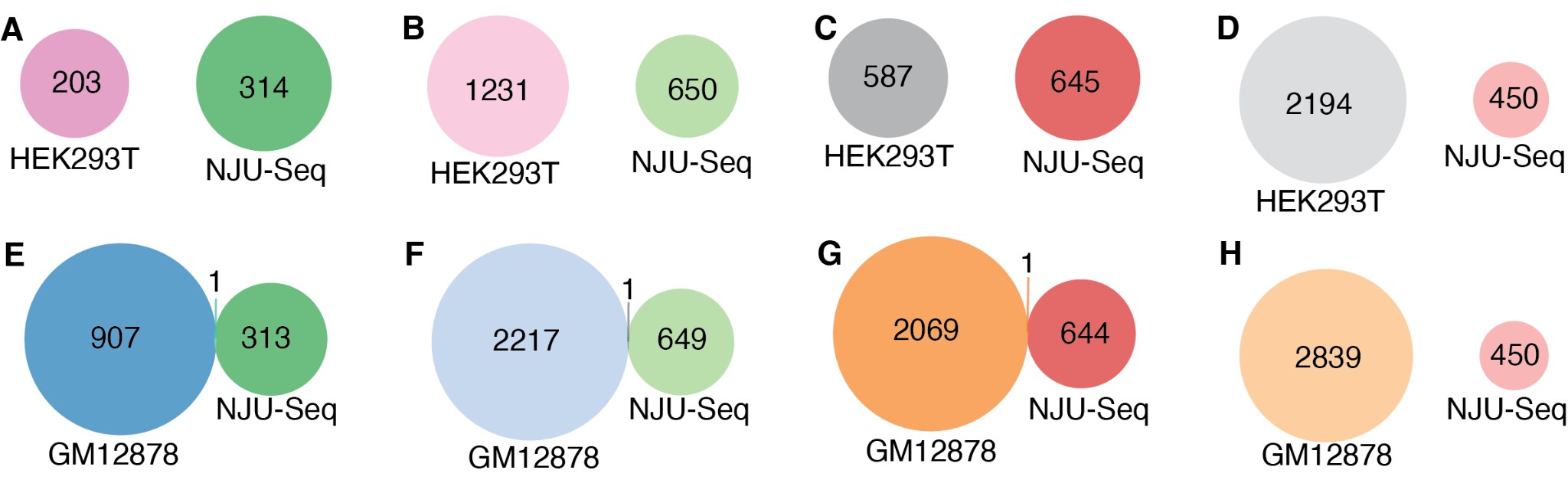 |
| --- |
| **Fig S10.** **2’ OMethyl Modification calling in HEK293 and GM12878 vs. RibOxi-Seq.** **A - D.** 2’OMethyl A, C, G and U from RibOxi-Seq versus HEK293T Dorado DRS modification called sites when filtered for 20 reads in coverage and 20% IVT-adjusted modification occupancy. **E - H.** 2’OMethyl A, C, G and U from RibOxi-Seq versus GM12878 Dorado DRS modification called sites when filtered for 20 reads in coverage and 20% IVT-adjusted modification occupancy. |

| 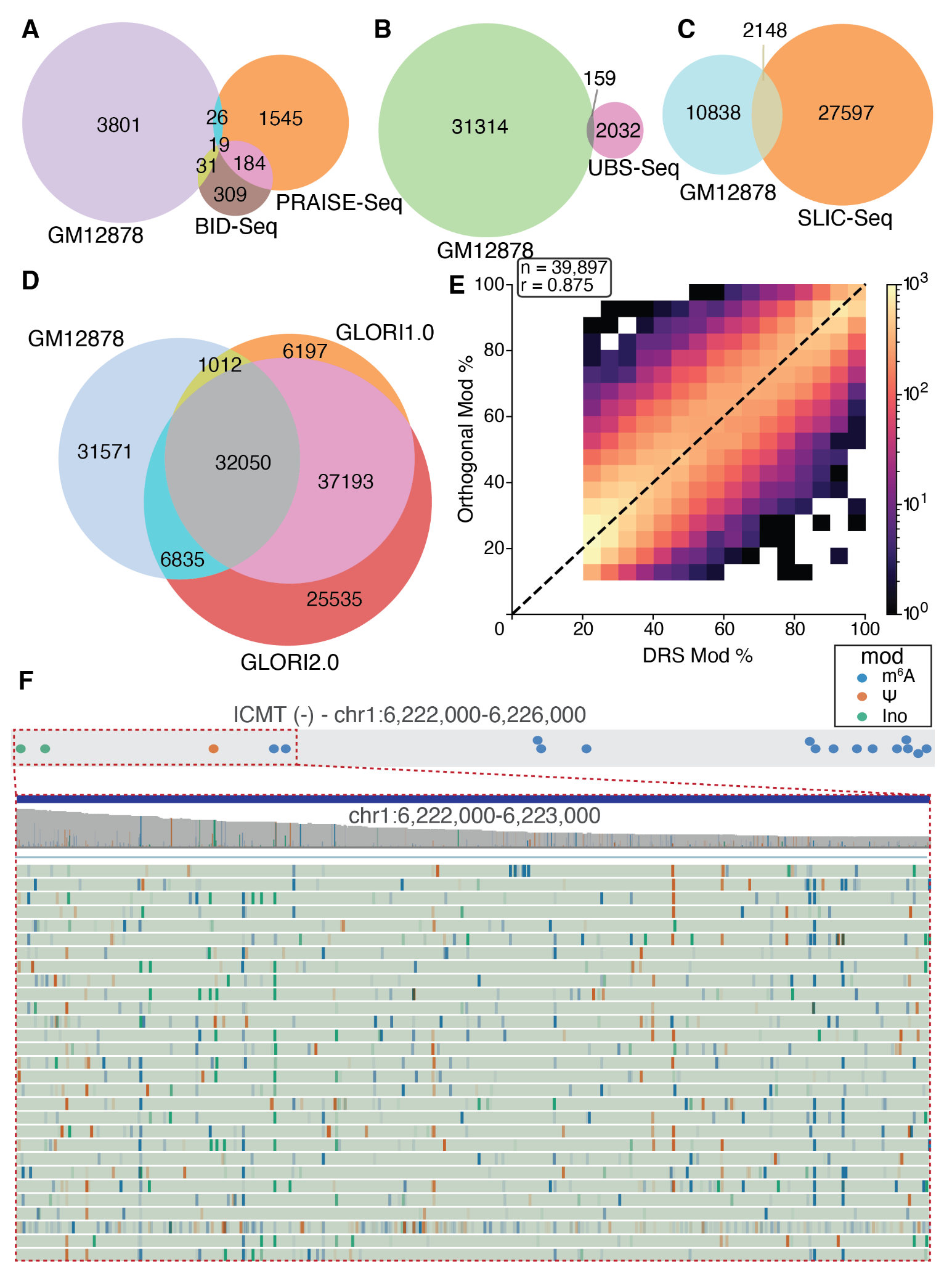 |
| --- |
| **Fig S11. Comparison of Dorado modification calling to orthogonal methods.** **A.** Dorado Ψ calling in GM12878 versus BID-Seq and PRAISE-Seq (HEK293). **B.** Dorado m^5^C calling in GM12878 vs. UBS-Seq (HEK293). **C.** Dorado inosine calling in GM12878 versus SLIC-Seq (HEK293). **D.** Dorado m^6^A calling in GM12878 vs. GLORI-Seq 1.0 and GLORI-Seq 2.0 chemistries (HEK293). **E.** Dorado m^6^A modification occupancy vs. combined GLORI-Seq modification occupancy. **F.** Exemplar gene ICMT exhibiting multiple modifications on it’s gene-body. |

| 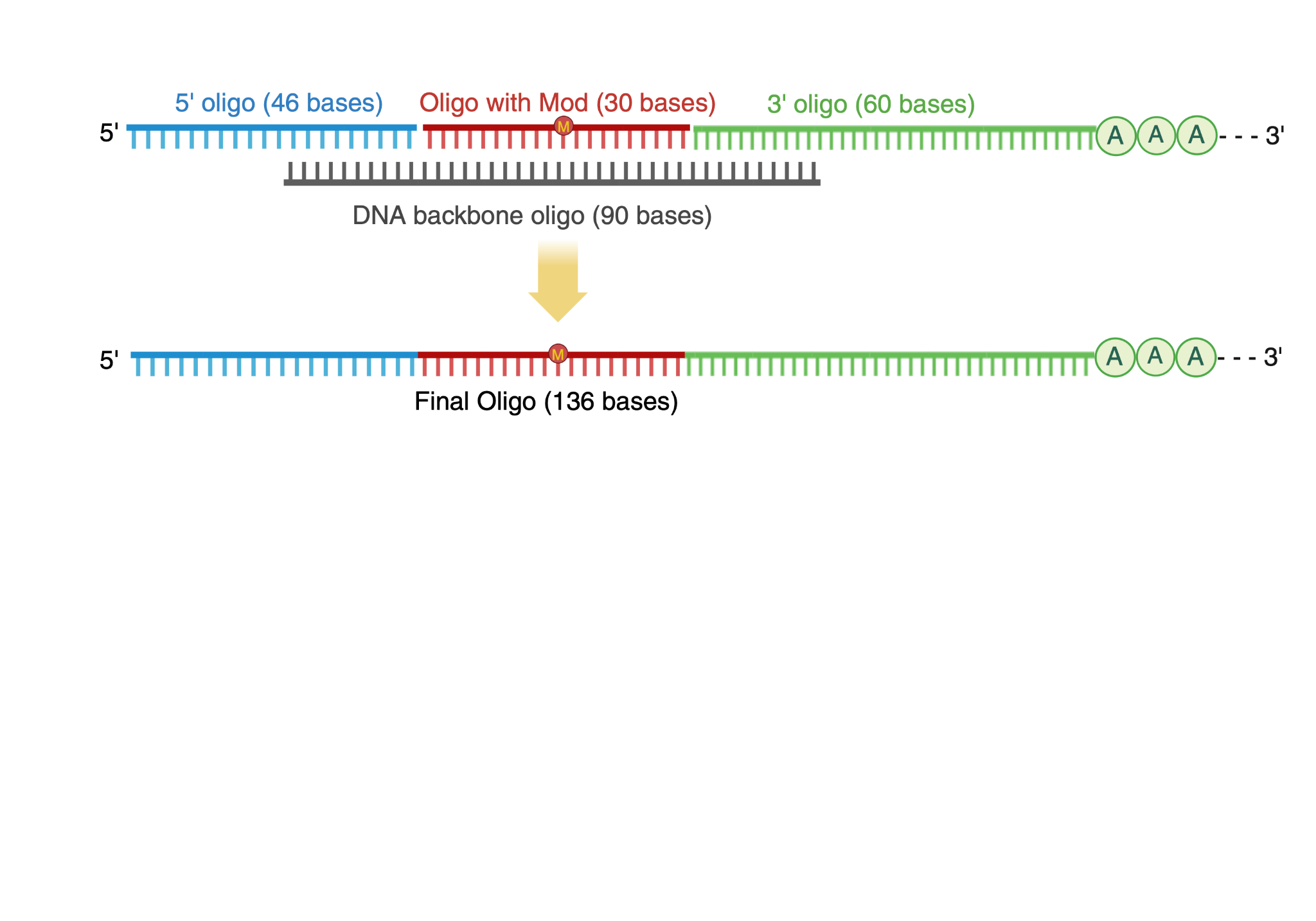 |
| --- |
| **Fig S12.** Schematic view of the splinted ligation strategy |
